# Technological Approach to Mind Everywhere: An Experimentally-Grounded Framework for Understanding Diverse Bodies and Minds

**DOI:** 10.3389/fnsys.2022.768201

**Published:** 2022-03-24

**Authors:** Michael Levin

**Affiliations:** ^1^Allen Discovery Center at Tufts University, Medford, MA, United States; ^2^Wyss Institute for Biologically Inspired Engineering at Harvard University, Cambridge, MA, United States

**Keywords:** regeneration, basal cognition, bioelectricity, gap junctions, synthetic morphology, bioengineering

## Abstract

Synthetic biology and bioengineering provide the opportunity to create novel embodied cognitive systems (otherwise known as minds) in a very wide variety of chimeric architectures combining evolved and designed material and software. These advances are disrupting familiar concepts in the philosophy of mind, and require new ways of thinking about and comparing truly diverse intelligences, whose composition and origin are not like any of the available natural model species. In this Perspective, I introduce TAME—Technological Approach to Mind Everywhere—a framework for understanding and manipulating cognition in unconventional substrates. TAME formalizes a non-binary (continuous), empirically-based approach to strongly embodied agency. TAME provides a natural way to think about animal sentience as an instance of collective intelligence of cell groups, arising from dynamics that manifest in similar ways in numerous other substrates. When applied to regenerating/developmental systems, TAME suggests a perspective on morphogenesis as an example of basal cognition. The deep symmetry between problem-solving in anatomical, physiological, transcriptional, and 3D (traditional behavioral) spaces drives specific hypotheses by which cognitive capacities can increase during evolution. An important medium exploited by evolution for joining active subunits into greater agents is developmental bioelectricity, implemented by pre-neural use of ion channels and gap junctions to scale up cell-level feedback loops into anatomical homeostasis. This architecture of multi-scale competency of biological systems has important implications for plasticity of bodies and minds, greatly potentiating evolvability. Considering classical and recent data from the perspectives of computational science, evolutionary biology, and basal cognition, reveals a rich research program with many implications for cognitive science, evolutionary biology, regenerative medicine, and artificial intelligence.

## Introduction

All known cognitive agents are collective intelligences, because we are all made of parts; biological agents in particular are not just structurally modular, but made of parts that are themselves agents in important ways. There is no truly monadic, indivisible yet cognitive being: all known minds reside in physical systems composed of components of various complexity and active behavior. However, as human adults, our primary experience is that of a centralized, coherent Self which controls events in a top-down manner. That is also how we formulate models of learning (“the *rat* learned X”), moral responsibility, decision-making, and valence: at the center is a subject which has agency, serves as the locus of rewards and punishments, possesses (as a single functional unit) memories, exhibits preferences, and takes actions. And yet, under the hood, we find collections of cells which follow low-level rules *via* distributed, parallel functionality and give rise to emergent system-level dynamics. Much as single celled organisms transitioned to multicellularity during evolution, the single cells of an embryo construct *de novo*, and then operate, a unified Self during a single agent’s lifetime. The compound agent supports memories, goals, and cognition that belongs to that Self and not to any of the parts alone. Thus, one of the most profound and far-reaching questions is that of scaling and unification: how do the activities of competent, lower-level agents give rise to a multiscale holobiont that is truly more than the sum of its parts? And, given the myriad of ways that parts can be assembled and relate to each other, is it possible to define ways in which truly diverse intelligences can be recognized, compared, and understood?

Here, I develop a framework to drive new theory and experiment in biology, cognition, evolution, and biotechnology from a multi-scale perspective on the nature and scaling of the cognitive Self. An important part of this research program is the need to encompass beings beyond the familiar conventional, evolved, static model animals with brains. The gaps in existing frameworks, and thus opportunities for fundamental advances, are revealed by a focus on plasticity of existing forms, and the functional diversity enabled by chimeric bioengineering. To illustrate how this framework can be applied to unconventional substrates, I explore a deep symmetry between behavior and morphogenesis, deriving hypotheses for dynamics that up- and down-scale Selves within developmental and phylogenetic timeframes, and at the same time strongly impact the speed of the evolutionary process itself (Dukas, 1998). I attempt to show how anatomical homeostasis can be viewed as the result of the behavior of the swarm intelligence of cells, and provides a rich example of how an inclusive, forward-looking technological framework can connect philosophical questions with specific empirical research programs.

The philosophical context for the following perspective is summarized in Table 1 (see also Glossary), and links tightly to the field of basal cognition (Birch et al., 2020) *via* a fundamentally gradualist approach. It should be noted that the specific proposals for biological mechanisms that scale functional capacity are synergistic with, but not linearly dependent on, this conceptual basis. The hypotheses about how bioelectric networks scale cell computation into anatomical homeostasis, and the evolutionary dynamics of multi-scale competency, can be explored without accepting the “minds everywhere” commitments of the framework. However, together they form a coherent lens onto the life sciences which helps generate testable new hypotheses and integrate data from several subfields.

**TABLE 1:** The core tenets of TAME.

• Continuum of cognitive capacities—no binary categories, no bright line separating true cognition from “just physics,” as is clear from evolutionary process and ability to bioengineer chimeras between any two “natural kinds.” • Mature frameworks must apply to truly diverse intelligences—beyond the examples from Earth’s phylogenetic tree based on brains, we must be able to consider and compare agents across the option space of designed and evolved combinations of living, non-living, and software components at all scales. • Selves exist across a continuum of persuadability, and it is an empirical question as to where on this axis any given system lies (revealed by the ratio of prediction and control vs. effort and knowledge that needs to be input, for any given way of relating to that system). • Selves are not fixed, permanent agents—their substrate can remodel radically during their lifetime; the owner of memories and preferences, and the subject that interprets rewards and punishments, is malleable and plastic. • The core of being a Self is the ability to pursue goals. Selves can be nested and overlapping, cooperating and competing both laterally and across levels. Each higher-level self deforms the option space for the lower level Selves, enabling them to follow energy minimization to achieve outcomes that look inevitable and simple at one scale, while serving intelligent goals at a higher scale. • Intelligence is the degree of competency of navigating any space (not just the familiar 3D space of motility), including morphospace, transcriptional space, physiological space, etc., toward desirable regions, while avoiding being trapped in local minima. Estimates of intelligence of any system are observer-dependent, and say as much about the observer and their limitations as they do about the system itself.

**FIGURE 1:**
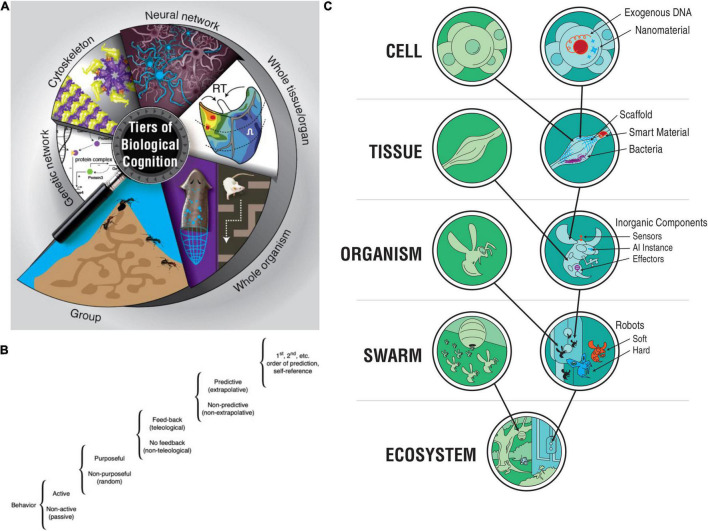
Diverse, multiscale intelligence. (A) Biology is organized in a multi-scale, nested architecture of molecular pathways. (B) These are not merely structural, but also computational: each level of this holarchy contains subsystems which exhibit some degree of problem-solving (i.e., intelligent) activity, on a continuum such as the one proposed by Rosenblueth et al. (1943). (C) At each layer of a given biosystem, novel components can be introduced of either biological or engineered origin, resulting in chimeric forms that have novel bodies and novel cognitive systems distinct from the typical model species on the Earth’s phylogenetic lineage. Images in panels (A,C) by Jeremy Guay of Peregrine Creative. Image in panel (B) was created after Rosenblueth et al. (1943).

For the purposes of this paper, “cognition” refers not only to complex, self-reflexive advanced cognition or metacognition, but is used in the less conservative sense that recognizes many diverse capacities for learning from experience (Ginsburg and Jablonka, 2021), adaptive responsiveness, self-direction, decision-making in light of preferences, problem-solving, active probing of their environment, and action at different levels of sophistication in conventional (evolved) life forms as well as bioengineered ones (Rosenblueth et al., 1943; Lyon, 2006; Bayne et al., 2019; Levin et al., 2021; Lyon et al., 2021; Figure 1). For our purposes, cognition refers to the functional computations that take place between perception and action, which allow the agent to span a wider range of time (*via* memory and predictive capacity, however much it may have) than its immediate *now*, which enable it to generalize and infer patterns from instances of stimuli—precursors to more advanced forms of recombining concepts, language, and logic.

**FIGURE 2:**
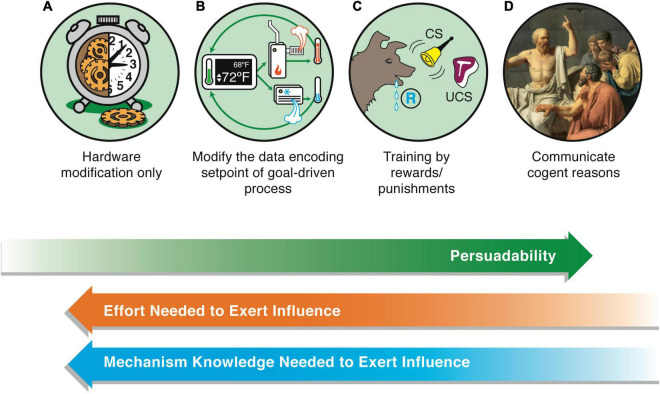
The axis of persuadability. A proposed way to visualize a continuum of agency, which frames the problem in a way that is testable and drives empirical progress, is *via* an “axis of persuadability”: to what level of control (ranging from brute force micromanagement to persuasion by rational argument) is any given system amenable, given the sophistication of its cognitive apparatus? Here are shown only a few representative waypoints. On the far left are the simplest physical systems, e.g., mechanical clocks (A). These cannot be persuaded, argued with, or even rewarded/punished—only physical hardware-level “rewiring” is possible if one wants to change their behavior. On the far right (D) are human beings (and perhaps others to be discovered) whose behavior can be radically changed by a communication that encodes a rational argument that changes the motivation, planning, values, and commitment of the agent receiving this. Between these extremes lies a rich panoply of intermediate agents, such as simple homeostatic circuits (B) which have setpoints encoding goal states, and more complex systems such as animals which can be controlled by signals, stimuli, training, etc., (C). They can have some degree of plasticity, memory (change of future behavior caused by past events), various types of simple or complex learning, anticipation/prediction, etc. Modern “machines” are increasingly occupying right-ward positions on this continuum (Bongard and Levin, 2021). Some may have preferences, which avails the experimenter of the technique of rewards and punishments—a more sophisticated control method than rewiring, but not as sophisticated as persuasion (the latter requires the system to be a logical agent, able to comprehend and be moved by arguments, not merely triggered by signals). Examples of transitions include turning the sensors of state outward, to include others’ stress as part of one’s action policies, and eventually the meta-goal of committing to enhance one’s agency, intelligence, or compassion (increase the scope of goals one can pursue). A more negative example is becoming sophisticated enough to be susceptible to a “thought that breaks the thinker” (e.g., existential or skeptical arguments that can make one depressed or even suicidal, Gödel paradoxes, etc.)—massive changes can be made in those systems by a very low-energy signal because it is treated as information in the context of a complex host computational machinery. These agents exhibit a degree of multi-scale plasticity that enables informational input to make strong changes in the structure of the cognitive system itself. The positive flip side of this vulnerability is that it avails those kinds of minds with a long term version of free will: the ability through practice and repeated effort to change their own thinking patterns, responses to stimuli, and functional cognition. This continuum is not meant to be a linear *scala naturae* that aligns with any kind of “direction” of evolutionary progress—evolution is free to move in any direction in this option space of cognitive capacity; instead, this scheme provides a way to formalize (for a pragmatic, engineering approach) the major transitions in cognitive capacity that can be exploited for increased insight and control. The goal of the scientist is to find the optimal position for a given system. Too far to the right, and one ends up attributing hopes and dreams to thermostats or simple AIs in a way that does not advance prediction and control. Too far to the left, and one loses the benefits of top-down control in favor of intractable micromanagement. Note also that this forms a continuum with respect to how much knowledge one has to have about the system’s details in order to manipulate its function: for systems in class A, one has to know a lot about their workings to modify them. For class B, one has to know how to read-write the setpoint information, but does not need to know anything about how the system will implement those goals. For class C, one doesn’t have to know how the system modifies its goal encodings in light of experience, because the system does all of this on its own—one only has to provide rewards and punishments. Images by Jeremy Guay of Peregrine Creative.

The framework, TAME—Technological Approach to Mind Everywhere—adopts a practical, constructive engineering perspective on the optimal place for a given system on the continuum of cognitive sophistication. This gives rise to an axis of *persuadability* (Figure 2), which is closely related to the Intentional Stance (Dennett, 1987) but made more explicit in terms of functional engineering approaches needed to implement prediction and control in practice. Persuadability refers to the type of conceptual and practical tools that are optimal to rationally modify a given system’s behavior. The origin story (designed vs. evolved), composition, and other aspects are not definitive guides to the correct level of agency for a living or non-living system. Instead, one must perform experiments to see which kind of intervention strategy provides the most efficient prediction and control (thus, one aim should be generalizing the human-focused Turing Test and other IQ metrics into a broader agency detection toolkit, which perhaps could itself be implemented by a useful algorithm).

Our capacity to find new ways to understand and manipulate complex systems is strongly related to how we categorize agency in our world. Newton didn’t invent two terms—gravity (for terrestrial objects falling) and perhaps *shmavity* (for the moon)—because it would have lost out on the much more powerful unification. TAME proposes a conceptual unification that would facilitate porting of tools across disciplines and model systems. We should avoid quotes around mental terms because there is no absolute, binary distinction between *it knows* and *it “knows”*—only a difference in the degree to which a model will be useful that incorporates such components.

Given this perspective, below I develop hypotheses about invariants that unify otherwise disparate-seeming problems, such as morphogenesis, behavior, and physiological allostasis. I take goals (in the cybernetic sense) and stressors (as a system-level result of distance from one’s goals) as key invariants which allow us to study and compare agents in truly diverse embodiments. The processes which scale goals and stressors form a positive feedback loop with modularity, thus both arising from, and potentiating the power of, evolution. These hypotheses suggest a specific way to understand the scaling of cognitive capacity through evolution, make interesting predictions, and suggest novel experimental work. They also provide ways to think about the impending expansion of the “space of possible bodies and minds” *via* the efforts of bioengineers, which is sure to disrupt categories and conclusions that have been formed in the context of today’s natural biosphere.

What of consciousness? It is likely impossible to understand sentience without understanding cognition, and the emphasis of this paper is on testable, empirical impacts of ways to understand cognition in all of its guises. By enabling the definition, detection, and comparison of cognition and intelligence, in diverse substrates beyond standard animals, we can enhance the range of embodiments in which sentience may result. In order to move the field forward *via* empirical progress, the focus of most of the discussion below is on ways to think about cognitive function, not on phenomenal or access consciousness [in the sense of the “Hard Problem” (Chalmers, 2013)]. However, I return to this issue at the end, discussing TAME’s view of sentience as fundamentally tied to goal-directed activity, only some aspects of which can be studied *via* third person approaches.

The main goal is to help advance and delineate an exciting emerging field at the intersection of biology, philosophy, and the information sciences. By proposing a new framework and examining it in a broad context of now physically realizable (not merely logically possible) living structures, it may be possible to bring conceptual, philosophical thought up to date with recent advances in science and technology. At stake are current knowledge gaps in evolutionary, developmental, and cell biology, a new roadmap for regenerative medicine, lessons that could be ported to artificial intelligence and robotics, and broader implications for ethics.

## Cognition: Changing the Subject

Even advanced animals are really collective intelligences (Couzin, 2007, 2009; Valentini et al., 2018), exploiting still poorly-understood scaling and binding features of metazoan architectures that share a continuum with looser swarms that have been termed “liquid brains” (Sole et al., 2019). Studies of “centralized control” focus on a brain, which is in effect a network of cells performing functions that many cell types, including bacteria, can do (Koshland, 1983). The embodied nature of cognition means that the minds of Selves are dependent on a highly plastic material substrate which changes not only on evolutionary time scales but also during the lifetime of the agent itself.

**FIGURE 3:**
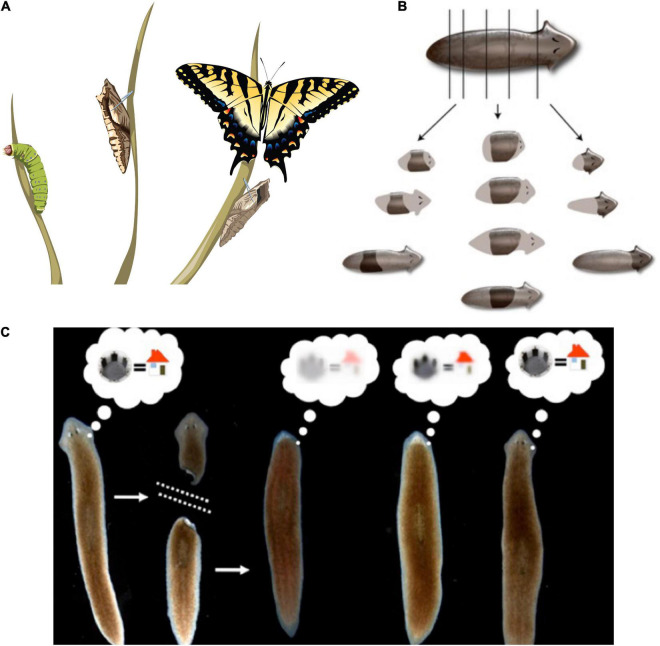
Cognitive Selves can change in real-time. (A) Caterpillars metamorphose into butterflies, going through a process in which their body, brain, and cognitive systems are drastically remodeled during the lifetime of a single agent. Importantly, memories remain and persist through this process (Blackiston et al., 2015). (B) Planaria cut into pieces regenerate, with each piece re-growing and remodeling precisely what is needed to form an entire animal. (C) Planarians derived from tail fragments of trained worms still retain original information, illustrating the ability of memories to move across tissues and be reimprinted on newly-developing brains (Corning, 1966, 1967; Shomrat and Levin, 2013). Images by Jeremy Guay of Peregrine Creative.

The central consequence of the composite nature of all intelligences is that the Self is subject to significant change in real-time (Figure 3). This means both slow maturation through experience (a kind of “software” change that doesn’t disrupt traditional ways of thinking about agency), as well as radical changes of the material in which a given mind is implemented (Levin, 2020). The owner, or subject of memories, preferences, and in more advanced cases, credit and blame, is very malleable. At the same time, fascinating mechanisms somehow ensure the persistence of Self (such as complex memories) despite drastic alterations of substrate. For example, the massive remodeling of the caterpillar brain, followed by the morphogenesis of an entirely different brain suitable for the moth or beetle, does not wipe all the memories of the larva but somehow maps them onto behavioral capacities in the post-metamorphosis host, despite its entirely different body (Alloway, 1972; Tully et al., 1994; Sheiman and Tiras, 1996; Armstrong et al., 1998; Ray, 1999; Blackiston et al., 2008). Not only that, but memories can apparently persist following the complete regeneration of brains in some organisms (McConnell et al., 1959; Corning, 1966; Shomrat and Levin, 2013) such as planaria, in which prior knowledge and behavioral tendencies are somehow transferred onto a newly-constructed brain. Even in vertebrates, such as fish (Versteeg et al., 2021) and mammals (von der Ohe et al., 2006), brain size and structure can change repeatedly during their lifespan. This is crucial to understanding agency and intelligence at multiple scales and in unfamiliar embodiments because observations like this begin to break down the notion of Selves as monadic, immutable objects with a privileged scale. Becoming comfortable with biological cognitive agents that are malleable in terms of form and function (change radically during the lifetime of an individual) makes it easier to understand the origins and changes of cognition during evolution or as the result of bioengineering effort.

This little-studied intersection between regeneration/remodeling and cognition highlights the fascinating plasticity of the body, brain, and mind; traditional model systems in which cognition is mapped onto a stable, discrete, mature brain are insufficient to fully understand the relationship between the Self and its material substrate. Many scientists study the behavioral properties of caterpillars, and of butterflies, but the transition zone in-between, from the perspective of philosophy of mind and cognitive science, provides an important opportunity to study the mind-body relationship by changing the body during the lifetime of the agent (not just during evolution). Note that continuity of being across drastic biological remodeling is not only relevant for unusual cases in the animal kingdom, but is a fundamental property of most life—even humans change from a collection of cells to a functional individual, *via* a gradual morphogenetic process that constructs an active Self in real time. This has not been addressed in biology, and likewise not yet in computer science, where machine learning approaches use static neural networks (there is not a formalism for altering artificial neural networks’ architecture on the fly).

What are the invariants that enable a Self to persist (and be recognizable by third-person investigations) despite such change? Memory is a good candidate (Shoemaker, 1959; Ameriks, 1976; Figure 3). However, at least certain kinds of memories can be transferred between individuals, by transplants of brain tissue or molecular engrams (Pietsch and Schneider, 1969; McConnell and Shelby, 1970; Bisping et al., 1971; Chen et al., 2014; Bedecarrats et al., 2018; Abraham et al., 2019). Importantly, the movement of memories across individual animals is only a special case of the movement of memory in biological tissue in general. Even when housed in the same “body,” memories must move between tissues—for example, in a trained planarian’s tail fragment re-imprinting its learned information onto the newly regenerated brain, or the movement of memories onto new brain tissue during metamorphosis. In addition to the spatial movement and re-mapping of memories onto new substrates, there is also a temporal component, as each memory is really an instance of communication between past and future Selves. The plasticity of biological bodies, made of cells that die, are born, and significantly rearrange their tissue architecture, suggests that the understanding of cognition is fundamentally a problem of collective intelligence: to understand how stable cognitive structures can persist and map onto swarm *dynamics*, with preferences and stressors that scale from those of their components.

This is applicable even to such a “stable” form as the human brain, which is often spoken of as a single Subject of experience and thought. First, the gulf between planarian regeneration/insect metamorphosis and human brains is going to be bridged by emerging therapeutics. It is inevitable that stem cell therapies for degenerative brain diseases (Forraz et al., 2013; Rosser and Svendsen, 2014; Tanna and Sachan, 2014) will confront us with humans whose brains are partially replaced by the naïve progeny of cells that were not present during the formation of memories and personality traits in the patient. Even prior to these advances, it was clear that phenomena such as dissociative identity disorder (Miller and Triggiano, 1992), communication with non-verbal brain hemispheres in commissurotomy patients (Nagel, 1971; Montgomery, 2003), conjoined twins with fused brains (Gazzaniga, 1970; Barilan, 2003), etc., place human cognition onto a continuous spectrum with respect to the plasticity of integrated Selves that reside within a particular biological tissue implementation.

Importantly, animal model systems are now providing the ability to harness that plasticity for functional investigations of the body-mind relationship. For example, it is now easy to radically modify bodies in a time-scale that is much faster than evolutionary change, to study the inherent plasticity of minds without eons of selection to shape them to fit specific body architectures. When tadpoles are created to have eyes on their tails, instead of their heads, they are still readily able to perform visual learning tasks (Blackiston and Levin, 2013; Blackiston et al., 2017). Planaria can readily be made with two (or more) brains in the same body (Morgan, 1904; Oviedo et al., 2010), and human patients are now routinely augmented with novel inputs [such as sensory substitution (Bach-y-Rita et al., 1969; Bach-y-Rita, 1981; Danilov and Tyler, 2005; Ptito et al., 2005)] or novel effectors, such as instrumentized interfaces allowing thought to control engineered devices such as wheelchairs in addition to the default muscle-driven peripherals of their own bodies (Green and Kalaska, 2011; Chamola et al., 2020; Belwafi et al., 2021). The central phenomenon here is plasticity: minds are not tightly bound to one specific underlying architecture (as most of our software is today), but readily mold to changes of genomic defaults. The logical extension of this progress is a focus on self-modifying living beings and the creation of new agents in which the mind:body system is simplified by entirely replacing one side of the equation with an engineered construct. The benefit would be that at least one half of the system is now well-understood.

For example, in hybrots, animal brains are functionally connected to robotics instead of their normal body (Reger et al., 2000; Potter et al., 2003; Tsuda et al., 2009; Ando and Kanzaki, 2020). It doesn’t even have to be an entire brain—a plate of neurons can learn to fly a flight simulator, and it lives in a new virtual world (DeMarse and Dockendorf, 2005; Manicka and Harvey, 2008; Beer, 2014), as seen from the development of closed-loop neurobiological platforms (Demarse et al., 2001; Potter et al., 2005; Bakkum et al., 2007b; Chao et al., 2008; Rolston et al., 2009a,b). These kinds of results are reminiscent of Philosophy 101’s “brain in a vat” experiment (Harman, 1973). Brains adjust to driving robots and other devices as easily as they adjust to controlling a typical, or highly altered, living body because minds are somehow adapted and prepared to deal with body alterations—throughout development, metamorphosis and regeneration, and evolutionary change.

The massive plasticity of bodies, brains, and minds means that a mature cognitive science cannot just concern itself with understanding standard “model animals” as they exist right now. The typical “subject,” such as a rat or fruit fly, which remains constant during the course of one’s studies and is conveniently abstracted as a singular Self or intelligence, obscures the bigger picture. The future of this field must expand to frameworks that can handle all of the possible minds across an immense option space of bodies. Advances in bioengineering and artificial intelligence suggest that we or our descendants will be living in a world in which Darwin’s “endless forms most beautiful” (this Earth’s *N* = 1 ecosystem outputs) are just a tiny sample of the true variety of possible beings. Biobots, hybrots, cyborgs, synthetic and chimeric animals, genetically and cellularly bioengineered living forms, humans instrumentized to knowledge platforms, devices, and each other—these technologies are going to generate beings whose body architectures are nothing like our familiar phylogeny. They will be a functional mix of evolved and designed components; at all levels, smart materials, software-level systems, and living tissue will be integrated into novel beings which function in their own exotic Umwelt. Importantly, the information that is used to specify such beings’ form and function is no longer only genetic—it is truly “epigenetic” because it comes not only from the creature’s own genome but also from human and non-human agents’ minds (and eventually, robotic machine-learning-driven platforms) that use cell-level bioengineering to generate novel bodies from genetically wild-type cells. In these cases, the genetics are no guide to the outcome (which highlights some of the profound reasons that genetics is hard to use to truly predict cognitive form and function even in traditional living species).

Now is the time to begin to develop ways of thinking about truly novel bodies and minds, because the technology is advancing more rapidly than philosophical progress. Many of the standard philosophical puzzles concerning brain hemisphere transplants, moving memories, replacing body/brain parts, etc. are now eminently doable in practice, while the theory of how to interpret the results lags. We now have the opportunity to begin to develop conceptual approaches to (1) understand beings without convenient evolutionary back-stories as explanations for their cognitive capacities (whose minds are created *de novo*, and not shaped by long selection pressures toward specific capabilities), and (2) develop ways to analyze novel Selves that are not amenable to simple comparisons with related beings, not informed by their phylogenetic position relative to known standard species, and not predictable from an analysis of their genetics. The implications range across insights into evolutionary developmental biology, advancing bioengineering and artificial life research, new roadmaps for regenerative medicine, ability to recognize exobiological life, and the development of ethics for relating to novel beings whose composition offers no familiar phylogenetic touchstone. Thus, here I propose the beginnings of a framework designed to drive empirical research and conceptual/philosophical analysis that will be broadly applicable to minds regardless of their origin story or internal architecture.

## Technological Approach to Mind Everywhere: A Proposal for a Framework

The Technological Approach to Mind Everywhere (TAME) framework seeks to establish a way to recognize, study, and compare truly diverse intelligences in the space of possible agents. The goal of this project is to identify deep invariants between cognitive systems of very different types of agents, and abstract away from inessential features such as composition or origin, which were sufficient heuristics with which to recognize agency in prior decades but will surely be insufficient in the future (Bongard and Levin, 2021). To flesh out this approach, I first make explicit some of its philosophical foundations, and then discuss specific conceptual tools that have been developed to begin the task of understanding embodied cognition in the space of mind-as-it-can-be (a sister concept to Langton’s motto for the artificial life community—“life as it can be”) (Langton, 1995).

### Philosophical Foundations of an Approach to Diverse Intelligences

One key pillar of this research program is the commitment to gradualism with respect to almost all important cognition-related properties: advanced minds are in important ways generated in a continuous manner from much more humble proto-cognitive systems. On this view, it is hopeless to look for a clear bright line that demarcates “true” cognition (such as that of humans, great apes, etc.) from metaphorical “as if cognition” or “just physics.” Taking evolutionary biology seriously means that there is a continuous series of forms that connect any cognitive system with much more humble ones. While phylogenetic history already refutes views of a magical arrival of “true cognition” in one generation, from parents that didn’t have it (instead stretching the process of cognitive expansion over long time scales and slow modification), recent advances in biotechnology make this completely implausible. For any putative difference between a creature that is proposed to have *true* preferences, memories, and plans and one that supposedly has *none*, we can now construct in-between, hybrid forms which then make it impossible to say whether the resulting being is an Agent or not. Many pseudo-problems evaporate when a binary view of cognition is dissolved by an appreciation of the plasticity and interoperability of living material at all scales of organization. A definitive discussion of the engineering of preferences and goal-directedness, in terms of hierarchy requirements and upper-directedness, is given in McShea (2013, 2016).

For example, one view is that only biological, evolved forms have intrinsic motivation, while software AI agents are only faking it *via* functional performance [but don’t actually *care* (Oudeyer and Kaplan, 2007, 2013; Lyon and Kuchling, 2021)]. But which biological systems *really* care—fish? Single cells? Do mitochondria (which used to be independent organisms) have true preferences about their own or their host cells’ physiological states? The lack of consensus on this question in classical (natural) biological systems, and the absence of convincing criteria that can be used to sort all possible agents to one or the other side of a sharp line, highlight the futility of truly binary categories. Moreover, we can now readily construct hybrid systems that consist of any percentage of robotics tightly coupled to on-board living cells and tissues, which function together as one integrated being. How many living cells does a robot need to contain before the living system’s “true” cognition bleeds over into the whole? On the continuum between human brains (with electrodes and a machine learning converter chip) that drive assistive devices (e.g., 95% human, 5% robotics), and robots with on-board cultured human brain cells instrumentized to assist with performance (5% human, 95% robotics), where can one draw the line—given that any desired percent combination is possible to make? No quantitative answer is sufficient to push a system “over the line” because there is no such line (at least, no convincing line has been proposed). Interesting aspects of agency or cognition are rarely if ever Boolean values.

Instead of a binary dichotomy, which leads to impassable philosophical roadblocks, we envision a continuum of advancement and diversity in information-processing capacity. Progressively more complex capabilities [such as unlimited associative learning, counterfactual modeling, symbol manipulation, etc., (Ginsburg and Jablonka, 2021)] ramp up, but are nevertheless part of a continuous process that is not devoid of proto-cognitive capacity before complex brains appear. Specifically, while major differences in cognitive function of course exist among diverse intelligences, transitions between them have not been shown to be binary or rapid relative to the timescale of individual agents. There is no plausible reason to think that evolution produces parents that don’t have “true cognition” but give rise to offspring that suddenly do, or that development starts with an embryo that has no “true preferences” and sharply transitions into an animal that does, etc. Moreover, bioengineering and chimerization can produce a smooth series of transitional forms between any two forms that are proposed to have, or not have, any cognitive property. Thus, agents gradually shift (during their lifetime, as result of development, metamorphosis, or interactions with other agents, or during evolutionary timescales) between great transitions in cognitive capacity, expressing and experiencing intermediate states of cognitive capacity that must be recognized by empirical approaches to study them.

A focus on the plasticity of the embodiments of mind strongly suggests this kind of gradualist view, which has been expounded in the context of evolutionary forces controlling individuality (Godfrey-Smith, 2009; Queller and Strassmann, 2009). Here the additional focus is on events taking place within the lifetime of individuals and driven by information and control dynamics. The TAME framework pushes experimenters to ask “how much” and “what kind of” cognition any given system might manifest if we interacted with it in the right way, at the right scale of observation. And of course, the degree of cognition is not a single parameter that gives rise to a *scala naturae* but a shorthand for the shape and size of its cognitive capacities in a rich space (discussed below).

The second pillar of TAME is that there is no privileged material substrate for Selves. Alongside familiar materials such as brains made of neurons, the field of basal cognition (Nicolis et al., 2011; Reid et al., 2012, 2013; Beekman and Latty, 2015; Baluška and Levin, 2016; Boussard et al., 2019; Dexter et al., 2019; Gershman et al., 2021; Levin et al., 2021; Lyon et al., 2021) has been identifying novel kinds of intelligences in single cells, plants, animal tissues, and swarms. The fields of active matter, intelligent materials, swarm robotics, machine learning, and someday, exobiology, suggest that we cannot rely on a familiar signature of “big vertebrate brain” as a necessary condition for mind. Molecular phylogeny shows that the specific components of brains pre-date the evolution of neurons *per se*, and life has been solving problems long before brains came onto the scene (Buznikov et al., 2005; Levin et al., 2006; Jekely et al., 2015; Liebeskind et al., 2015; Moran et al., 2015). Powerful unification and generalization of concepts from cognitive science and other fields can be achieved if we develop tools to characterize and relate to a wide diversity of minds in unconventional material implementations (Damasio, 2010; Damasio and Carvalho, 2013; Cook et al., 2014; Ford, 2017; Man and Damasio, 2019; Baluska et al., 2021; Reber and Baluska, 2021).

Closely related to that is the de-throning of natural evolution as the only acceptable origin story for a true Agent [many have proposed a distinction between evolved living forms vs. the somehow inadequate machines which were merely designed by man (Bongard and Levin, 2021)]. First, synthetic evolutionary processes are now being used in the lab to create “machines” and modify life (Kriegman et al., 2020a; Blackiston et al., 2021). Second, the whole process of evolution, basically a hill-climbing search algorithm, results in a set of frozen accidents and meandering selection among random tweaks to the micro-level hardware of cells, with impossible to predict large-scale consequences for the emergent system level structure and function. If this short-sighted process, constrained by many forces that have nothing to do with favoring complex cognition, can give rise to true minds, then so can a rational engineering approach. There is nothing magical about evolution (driven by randomizing processes) as a forge for cognition; surely we can eventually do at least as well, and likely much better, using rational construction principles and an even wider range of materials.

The third foundational aspect of TAME is that the correct answer to how much agency a system has cannot be settled by philosophy—it is an empirical question. The goal is to produce a framework that drives experimental research programs, not only philosophical debate about what should or should not be possible as a matter of definition. To this end, the productive way to think about this a variant of Dennett’s Intentional Stance (Dennett, 1987; Mar et al., 2007), which frames properties such as cognition as observer-dependent, empirically testable, and defined by how much benefit their recognition offers to science (Figure 2). Thus, the correct level of agency with which to treat any system must be determined by experiments that reveal which kind of model and strategy provides the most efficient predictive and control capability over the system. In this engineering (understand, modify, build)-centered view, the optimal position of a system on the spectrum of agency is determined empirically, based on which kind of model affords the most efficient way of prediction and control. Such estimates are, by their empirical nature, subject to revision by future experimental data and conceptual frameworks, and are observer-dependent (not absolute).

A standard methodology in science is to avoid attributing agency to a given system unless absolutely necessary. The mainstream view (e.g., Morgan’s Canon) is that it’s too easy to fall into a trap of “anthropomorphizing” systems with only apparent cognitive powers, when one should only be looking for models focused on mechanistic, lower levels of description that eschew any kind of teleology or mental capacity (Morgan, 1903; Epstein, 1984). However, analysis shows that this view provides no useful parsimony (Cartmill, 2017). The rich history of debates on reductionism and mechanism needs to be complemented with an empirical, engineering approach that is not inappropriately slanted in one direction on this continuum. Teleophobia leads to Type 2 errors with respect to attribution of cognition that carry a huge opportunity cost for not only practical outcomes like regenerative medicine (Pezzulo and Levin, 2015) and engineering, but also ethics. Humans (and many other animals) readily attribute agency to systems in their environment; scientists should be comfortable with testing out a theory of mind regarding various complex systems for the exact same reason—it can often greatly enhance prediction and control, by recognizing the true features of the systems with which we interact. This perspective implies that there is no such thing as “anthropomorphizing” because human beings have no unique essential property which can be inappropriately attributed to agents that have *none* of it. Aside from the very rare trivial cases (misattributing human*-level* cognition to simpler systems), we must be careful to avoid the pervasive, implicit remnants of a human-centered pre-scientific worldview in which modern, standard humans are assumed to have some sort of irreducible quality that cannot be present in degrees in slightly (or greatly) different physical implementations (from early hominids to cyborgs etc.). Instead, we should seek ways to naturalize human capacities as elaborations of more fundamental principles that are widely present in complex systems, in very different types and degrees, and to identify the *correct* level for any given system. Of course, this is just one stance, emphasizing experimental, not philosophical, approaches that avoid defining impassable absolute differences that are not explainable by any known binary transition in body structure or function. Others can certainly drive empirical work focused specifically on what kind of human-level capacities do and do not exist in detectable quantity in other agents.

Avoiding philosophical wrangling over privileged levels of explanation (Ellis, 2008; Ellis et al., 2012; Noble, 2012), TAME takes an empirical approach to attributing agency, which increases the toolkit of ways to relate to complex systems, and also works to reduce profligate attributions of mental qualities. We do not say that a thermos knows whether to keep something hot or cold, because no model of thermos cognition does better than basic thermodynamics to explain its behavior or build better thermoses. At the same time, we know we cannot simply use Newton’s laws to predict the motion of a (living) mouse at the top of a hill, requiring us to construct models of navigation and goal-directed activity for the controller of the mouse’s behavior over time (Jennings, 1906).

Under-estimating the capacity of a system for plasticity, learning, having preferences, representation, and intelligent problem-solving greatly reduces the toolkit of techniques we can use to understand and control its behavior. Consider the task of getting a pigeon to correctly distinguish videos of dance vs. those of martial arts. If one approaches the system bottom-up, one has to implement ways to interface to individual neurons in the animal’s brain to read the visual input, distinguish the videos correctly, and then control other neurons to force the behavior of walking up to a button and pressing it. This may someday be possible, but not in our lifetimes. In contrast, one can simply train the pigeon (Qadri and Cook, 2017). Humanity has been training animals for millennia, without knowing anything about what is in their heads or how brains work. This highly efficient trick works because we correctly identified them as learning agents, which allows us to offload a lot of the computational complexity of any task onto the living system itself, without micromanaging its components.

What other systems might this remarkably powerful strategy apply to? For example, gene regulatory networks (GRNs) are a paradigmatic example of “genetic mechanism,” often assumed to be tractable only by hardware (requiring gene therapy approaches to alter promoter sequences that control network connectivity, or adding/removing gene nodes). However, being open to the possibility that GRNs might actually be on a different place on this continuum suggests an experiment in which they are trained for new behaviors with specific combinations of stimuli (experiences). Indeed, recent analyses of biological GRN models reveal that they exhibit associative and several other kinds of learning capacity, as well as pattern completion and generalization (Watson et al., 2010, 2014; Szilagyi et al., 2020; Biswas et al., 2021). This is an example in which an empirical approach to the correct level of agency for even simple systems not usually thought of as cognitive suggests new hypotheses which in turn open a path to new practical applications (biomedical strategies using associative regimes of drug pulsing to exploit memory and address pharmacoresistance by abrogating habituation, etc.).

We next consider specific aspects of the framework, before diving into specific examples in which it drives novel empirical work.

### Specific Conceptual Components of the Technological Approach to Mind Everywhere Framework

A useful framework in this emerging field should not only serve as a lens with which to view data and concepts (Manicka and Levin, 2019b), but also should drive research in several ways. It needs to first specify definitions for key terms such as a Self. These are not meant to be exclusively correct—the definitions can co-exist with others, but should identify a claim as to what is an essential invariant for Selves (and what other aspects can diverge), and how it intersects with experiment. The fundamental symmetry unifying all possible Selves should also facilitate direct comparison or even classification of truly diverse intelligences, sketching the markers of Selfhood and the topology of the option space within which possible agents exist. The framework should also help scientists derive testable claims about how borders of a given Self are determined, and how it interacts with the outside world (and other agents). Finally, the framework should provide actionable, semi-quantitative definitions that have strong implications and constrain theories about how Selves arise and change. All of this must facilitate experimental approaches to determine the empirical utility of this approach.

The TAME framework takes the following as the basic hallmarks of being a Self: the ability to pursue goals, to own compound (e.g., associative) memories, and to serve as the locus for credit assignment (be rewarded or punished), where all of these are at a scale larger than possible for any of its components alone. Given the gradualist nature of the framework, the key question for any agent is “how well,” “how much,” and “what kind” of capacity it has for each of those key aspects, which in turn allows agents to be directly compared in an option space. TAME emphasizes defining a higher scale at which the (possibly competent) activity of component parts gives rise to an emergent system. Like a valid mathematical theorem which has a unique structure and existence over and above any of its individual statements, a Self can own, for example, associative memories (that bind into new mental content experiences that occurred separately to its individual parts), be the subject of reward or punishment for complex states (as a consequence of highly diverse actions that its parts have taken), and be stressed by states of affairs (deviations from goals or setpoints) that are not definable at the level of any of its parts (which of course may have their own distinct types of stresses and goals). These are practical aspects that suggest ways to recognize, create, and modify Selves.

**FIGURE 4:**
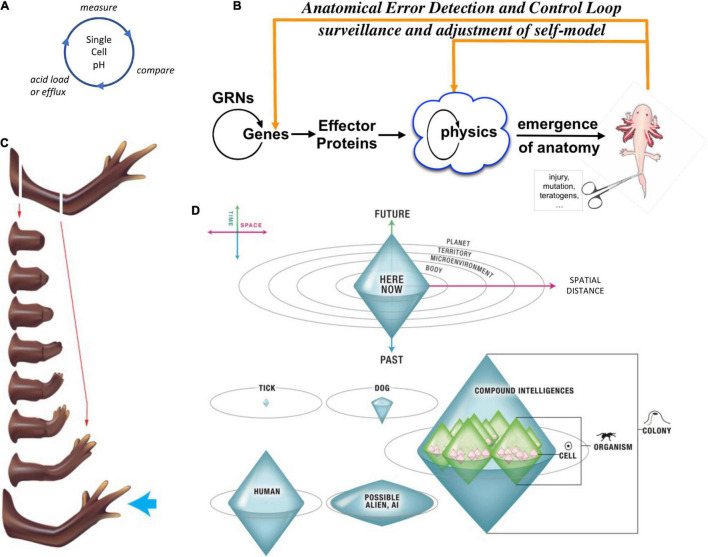
Unconventional goal-directed agents and the scaling of the cognitive Self. (A) The minimal component of agency is homeostasis, for example the ability of a cell to execute the Test-Operate-Exit (Pezzulo and Levin, 2016) loop: a cycle of comparison with setpoint and adjustment *via* effectors, which allows it to remain in a particular region of state space. (B) This same capacity is scaled up by cellular networks into anatomical homeostasis: morphogenesis is not simply a feedforward emergent process but rather the ability of living systems to adjust and remodel to specific target morphologies. This requires feedback loops at the transcriptional and biophysical levels, which rely on stored information (e.g., bioelectrical pattern memories) against which to minimize error. (C) This is what underlies complex regeneration such as salamander limbs, which can be cut at any position and result in just the right amount and type of regenerative growth that stops when a correct limb is achieved. Such homeostatic systems are examples of simple goal-directed agents. (D) A focus on the size or scale of goals any given system can pursue allows plotting very diverse intelligences on the same graph, regardless of their origin or composition (Levin, 2019). The scale of their goal-directed activity is estimated (collapsed onto one axis of space and one of time, as in Relativity diagrams). Importantly, this way of visualizing the sophistication of agency is a schematic of goal space—it is not meant to represent the spatial extent of sensing or effector range, but rather the scale of events about which they care and the boundary of states that they can possibly represent or work to change. This defines a kind of cognitive light cone (a boundary to any agent’s area of concern); the largest area represents the “now,” with fading efficacy both backward (accessing past events with decreasing reliability) and forward (limited prediction accuracy for future events). Agents are compound entities, composed of (and comprising) other sub- or super-agents each of which has their own cognitive boundary of various sizes. Images by Jeremy Guay of Peregrine Creative.

Selves can be classified and compared with respect to the scale of goals they can pursue [Figure 4, described in detail in Levin (2019)]. In this context, the goal-directed perspective adopted here builds on the work of Rosenblueth et al. (1943); Nagel (1979); and Mayr (1992), emphasizing plasticity (ability to reach a goal state from different starting points) and persistence (capacity to reach a goal (Schlosser, 1998) state despite perturbations).

The ability of a system to exert energy to work toward a state of affairs, overcoming obstacles (to the degree that its sophistication allows) to achieve a particular set of substates is very useful for defining Selves because it grounds the question in well-established control theory and cybernetics (i.e., systems “trying to do things” is no longer magical but is well-established in engineering), and provides a natural way of discovering, defining, and altering the preferences of a system. A common objection is: “surely we can’t say that thermostats have goals and preferences?” The TAME framework holds that whatever true goals and preferences are, there must exist primitive, minimal versions from which they evolved and these are, in an important sense, substrate- and scale-independent; simple homeostatic circuits are an ideal candidate for the “hydrogen atom” of goal-directed activity (Rosenblueth et al., 1943; Turner, 2019). A key tool for thinking about these problems is to ask what a truly minimal example of any cognitive capacity would be like, and to think about transitional forms that can be created just below that. It is logically inevitable that if one follows a complex cognitive capacity backward through phylogeny, one eventually reaches precursor versions of that capacity that naturally suggest the (misguided) question “is that *really* cognitive, or just physics?” Indeed, a kind of minimal goal-directedness permeates all of physics (Feynman, 1942; Georgiev and Georgiev, 2002; Ogborn et al., 2006; Kaila and Annila, 2008; Ramstead et al., 2019; Kuchling et al., 2020a), supporting a continuous climb of the scale and sophistication of goals.

Pursuit of goals is central to composite agency and the “many to one” problem because it requires distinct mechanisms (for measurement of states, storing setpoints, and driving activity to minimize the delta between the former and the latter) to be bound together into a functional unit that is greater than its parts. To co-opt a great quote (Dobzhansky, 1973), nothing in biology makes sense except in light of teleonomy (Pittendrigh, 1958; Nagel, 1979; Mayr, 1992; Schlosser, 1998; Noble, 2010, 2011; Auletta, 2011; Ellis et al., 2012). The degree to which a system can evaluate possible consequences of various actions, in pursuit of those goal states, can vary widely, but is essential to its survival. The expenditure of energy *in ways that effectively reach specific states despite uncertainty, limitations of capability, and meddling from outside forces* is proposed as a central unifying invariant for all Selves—a basis for the space of possible agents. This view suggests a semi-quantitative multi-axis option space that enables direct comparison of diverse intelligences of all sorts of material implementation and origins (Levin, 2019, 2020). Specifically (Figure 4), a “space-time” diagram can be created where the spatio-temporal *scale* of any agent’s goals delineates that Self and its cognitive boundaries.

Note that the distances on Figure 4D represent not first-order capacities such as sensory perception (how far away can it sense), but second-order capacities of the size of goals (humble metabolic hunger-satiety loops or grandiose planetary-scale engineering ambitions) which a given cognitive system is capable of representing and working toward. At any given time, an Agent is represented by a single shape in this space, corresponding to the size and complexity of their possible goal domain. However, genomes (or engineering design specs) map to an ensemble of such shapes in this space because the borders between Self and world, and the scope of goals an agent’s cognitive apparatus can handle, can all shift during the lifetime of some agents—“in software” (another “great transition” marker). All regions in this space can potentially define some possible agent. Of course, additional subdivisions (dimensions) can easily be added, such as the Unlimited Associative Learning marker (Birch et al., 2020) or aspects of Active Inference (Friston and Ao, 2012; Friston et al., 2015b; Calvo and Friston, 2017; Peters et al., 2017).

Some agents, like microbes, have minimal memory (Vladimirov and Sourjik, 2009; Lan and Tu, 2016) and can concern themselves only with a very short time horizon and spatial radius—e.g., follow local gradients. Some agents, e.g., a rat have more memory and some forward planning ability (Hadj-Chikh et al., 1996; Raby and Clayton, 2009; Smith and Litchfield, 2010), but are still precluded from, for example, effectively caring about what will happen 2 months hence, in an adjacent town. Some, like human beings, can devote their lives to causes of enormous scale (future state of the planet, humanity, etc.). Akin to Special Relativity, this formalization makes explicit that class of capacities (in terms of representation of classes of goals) that are forever inaccessible to a given agent (demarcating the edge of the “light cone” of its cognition).

In general, larger selves (1) are capable of working toward states of affairs that occur farther into the future (perhaps outlasting the lifetime of the agent itself—an important great transition, in the sense of West et al. (2015), along the cognitive continuum); (2) deploy memories further back in time (their actions become less “mechanism” and more *decision-making* (Balazsi et al., 2011) because they are linked to a network of functional causes and information with larger diameter); and (3) they expend effort to manage sensing/effector activity in larger spaces [from subcellular networks to the extended mind (Clark and Chalmers, 1998; Turner, 2000; Timsit and Gregoire, 2021)]. Overall, increases of agency are driven by mechanisms that scale up stress (Box 1)—the scope of states that an agent can possibly be stressed about (in the sense of pressure to take corrective action). In this framework, stress (as a system-level response to distance from setpoint states), preferences, motivation, and the ability to functionally care about what happens are tightly linked. Homeostasis, necessary for life, evolves into allostasis (McEwen, 1998; Schulkin and Sterling, 2019) as new architectures allow tight, local homeostatic loops to be scaled up to measure, cause, and remember larger and more complex states of affairs (Di Paulo, 2000; Camley, 2018).

**BOX 1. Stress as the glue of agency**

Tell me what you are stressed about and I will know a lot about your cognitive sophistication. Local glucose concentration? Limb too short? Rival is encroaching on your territory? Your limited lifespan? Global disparities in quality of life on Earth? The scope of states that an agent can possibly be stressed by, in effect, defines their degree of cognitive capacity. Stress is a systemic response to a difference between current state and a desired setpoint; it is an essential component to scaling of Selves because it enables different modules (which sense and act on things at different scales and in distributed locations) to be bound together in one global homeostatic loop (toward a larger purpose). Systemic stress occurs when one sub-agent is not satisfied about its local conditions, and propagates its unhappiness outward as hard-to-ignore signals. In this process, stress pathways serve the same function as hidden layers in a network, enabling the system to be more adaptive by connecting diverse modular inputs and outputs to the same basic stress minimization loop. Such networks scale stress, but stress is also what helps the network scale up its agency—a bidirectional positive feedback loop.

The key is that this stress signal is unpleasant to the other sub-agents, closely mimicking their own stress machinery (genetic conservation: my internal stress molecule is the same as your stress molecule, which contributes to the same “wiping of ownership” that is implemented by gap junctional connections). By propagating unhappiness in this way (in effect, turning up the global system “energy” which facilitates tendency for moving in various spaces), this process recruits distant sub-agents to act, to reduce their own perception of stress. For example, if an organ primordium is in the wrong location and needs to move, the surrounding cells are more willing to get out of the way if by doing so they reduce the amount of stress signal they receive. It may be a process akin to run-and-tumble for bacteria, with stress as the indicator of when to move and when to stop moving, in physiological, transcriptional, or morphogenetic space. Another example is compensatory hypertrophy, in which damage in one organ induces other cells to take up its workload, growing or taking on new functions if need be (Tamori and Deng, 2014; Fontes et al., 2020). In this way, stress causes other agents to work toward the same goal, serving as an influence that binds subunits across space into a coherent higher Self and resists the “struggle of the parts” (Heams, 2012). Interestingly, stress spreads not only horizontally in space (across cell fields) but also vertically, in time: effects of stress response is one of the things most easily transferred by transgenerational inheritance (Xue and Acar, 2018).

Additional implications of this view are that Selves: are malleable (the borders and scale of any Self can change over time); can be created by design or by evolution; and are multi-scale entities that consist of other, smaller Selves (and conversely, scale up to make larger Selves). Indeed they are a patchwork of agents [akin to Theophile Bordeu’s “many little lives” (Haigh, 1976; Wolfe, 2008)] that overlap with each other, and compete, communicate, and cooperate both horizontally (at their own level of organization) and vertically [with their component subunits and the super-Selves of which they are a part (Sims, 2020)].

**FIGURE 5:**
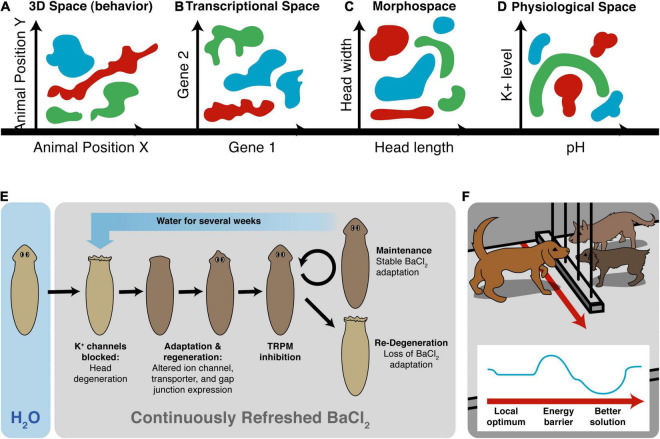
Cognitive agents solve problems in diverse spaces. Intelligence is fundamentally about problem-solving, but this takes place not only in familiar 3D space as “behavior” (control of muscle effectors for movement) (A), but also in other spaces in which cognitive systems try to navigate, in order to reach better regions. This includes the transcriptional space of gene expression (B) here schematized for two genes, anatomical morphospace (C) here schematized for two traits, and physiological space (D) here schematized for two parameters. An example (E) of problem-solving is planaria, which placed in barium (causing their heads to explode due to general blockade of potassium channels) regenerate new heads that are barium-insensitive (Emmons-Bell et al., 2019). They solve this entirely novel (not primed by evolutionary experience with barium) stressor by a very efficient traversal in transcriptional space to rapidly up/down regulate a very small number of genes that allows them to conduct their physiology despite the essential K^+^ flux blockade. (F) The degree of intelligence of a system can be estimated by how effectively they navigate to optimal regions without being caught in a local maximum, illustrated as a dog which could achieve its goal on the other side of the fence, but this would require going around—temporarily getting further from its goal (a measurable degree of patience or foresight of any system in navigating its space, which can be visualized as a sort of energy barrier in the space, inset). Images by Jeremy Guay of Peregrine Creative.

Another important invariant for comparing diverse intelligences is that they are all solving problems, in some space (Figure 5). It is proposed that the traditional problem-solving behavior we see in standard animals in 3D space is just a variant of evolutionarily more ancient capacity to solve problems in metabolic, physiological, transcriptional, and morphogenetic spaces (as one possible sequential timeline along which evolution pivoted some of the same strategies to solve problems in new spaces). For example, when planaria are exposed to barium, a non-specific potassium channel blocker, their heads explode. Remarkably, they soon regenerate heads that are completely insensitive to barium (Emmons-Bell et al., 2019). Transcriptomic analysis revealed that relatively few genes out of the entire genome were regulated to enable the cells to resolve this physiological stressor using transcriptional effectors to change how ions and neurotransmitters are handled by the cells. Barium is not something planaria ever encounter ecologically (so there should not be innate evolved responses to barium exposure), and cells don’t turn over fast enough for a selection process (e.g., with bacterial persisters after antibiotic exposure). The task of determining which genes, out of the entire genome, can be transcriptionally regulated to return to an appropriate physiological regime is an example of an unconventional intelligence navigating a large-dimensional space to solve problems in real-time (Voskoboynik et al., 2007; Elgart et al., 2015; Soen et al., 2015; Schreier et al., 2017). Also interesting is that the actions taken in transcriptional space (a set of mRNA states) map onto a path in physiological state (the *ability* to perform many needed functions despite abrogated K^+^ channel activity, not just a single state).

The common feature in all such instances is that the agent must navigate its space(s), preferentially occupying adaptive regions despite perturbations from the outside world (and from internal events) that tend to pull it into novel regions. Agents (and their sub- and super-agents) construct internal models of their spaces (Beer, 2014, 2015; Beer and Williams, 2015; Hoffman et al., 2015; Fields et al., 2017; Hoffman, 2017; Prentner, 2019; Dietrich et al., 2020; Prakash et al., 2020), which may or may not match the view of their action space developed by their conspecifics, parasites, and scientists. Thus, the space one is navigating is in an important sense virtual (belonging to some Agent’s self-model), is developed and often modified “on the fly” (in addition to that hardwired by the structure of the agent), and not only faces outward to infer a useful structure of its option space but also faces inward to map its own body and somatotopic properties (Bongard et al., 2006). The lower-level subsystems simplify the search space for the higher-level agent because their modular competency means that the higher-level system doesn’t need to manage all the microstates [a strong kind of hierarchical modularity (Zhao et al., 2006; Lowell and Pollack, 2016)]. In turn, the higher-level system deforms the option space for the lower-level systems so that they do not need to be as clever, and can simply follow local energy gradients.

The degree of intelligence, or sophistication, of an agent in any space is roughly proportional to its ability to deploy memory and prediction (information processing) in order to avoid local maxima. Intelligence involves being able to temporarily move away from a simple vector toward one’s goals in a way that results in bigger improvements down the line; the agent’s internal complexity has to facilitate some degree of complexity (akin to hidden layers in an artificial neural network which introduce plasticity between stimulus and response) in the goal-directed activity that enables the buffering needed for patience and indirect paths to the goal. This buffering enables the flip side of homeostatic problem-driven (stress reduction) behavior by cells: the exploration of the space for novel opportunities (creativity) by the collective agent, and the ability to acquire more complex goals [in effect, beginning the climb to Maslow’s hierarchy (Taormina and Gao, 2013)]. Of course it must be pointed out that this way of conceiving intelligence is one of many, and is proposed here as a way to enable the concept to be experimentally ported over to unfamiliar substrates, while capturing what is essential about it in a way that does not depend on arbitrary restrictions that will surely not survive advances in synthetic bioengineering, machine learning, and exobiology.

Another important aspect of intelligence that is space-agnostic is the capacity for generalization. For example, in the barium planaria example discussed above, it is possible that part of the problem-solving capacity is due to the cells’ ability to generalize in physiological space. Perhaps the cells recognize the physiological stresses induced by the novel barium stimulus as a member of the wider class of excitotoxicity induced by evolutionarily-familiar epileptic triggers, enabling them to deploy similar solutions (in terms of actions in transcriptional space). Such abilities to generalize have now been linked to measurement invariance (Frank, 2018), showing its ancient roots in the continuum of cognition.

Consistent with the above discussion, complex agents often consist of components that are themselves competent problem-solvers in their own (usually smaller, local) spaces. The relationship between wholes and their parts can be as follows. An agent is an integrated holobiont to the extent that it distorts the option space, and the geodesics through it, for its subunits (perhaps akin to how matter and space affect each other in general relativity) to get closer to a high-level goal in its space. A similar scheme is seen in neuroscience, where top-down feedback helps lower layer neurons to choose a response to local features by informing them about more global features (Krotov, 2021).

At the level of the subunits, which know nothing of the higher problem space, this simply looks like they are minimizing free energy and passively doing the only thing they can do as physical systems: this is why if one zooms in far enough on any act of decision-making, all one ever sees is dumb mechanism and “just physics.” The agential perspective (Godfrey-Smith, 2009) looks different at different scales of observation (and its degree is in the eye of a beholder who seeks to control and predict the system, which includes the Agent itself, and its various partitions). This view is closely aligned with that of “upper directedness” (McShea, 2012), in which the larger system directs its components’ behavior by constraints and rewards for coarse-grained outcomes, not microstates (McShea, 2012).

Note that these different competing and cooperating partitions are not just diverse components of the body (cells, microbiome, etc.) but also future and past versions of the Self. For example, one way to achieve the goal of a healthier metabolism is to lock the refrigerator at night and put the keys somewhere that your midnight self, which has a shorter cognitive boundary (is willing to trade long-term health for satiety right now) and less patience, is too lazy to find. Changing the option space, energy barriers, and reward gradients for your future self is a useful strategy for reaching complex goals despite the shorter horizons of the other intelligences that constitute your affordances in action space.

The most effective collective intelligences operate by simultaneously distorting the space to make it easy for their subunits to do the right thing with no comprehension of the larger-scale goals, but themselves benefit from the competency of the subunits which can often get their local job done even if the space is not perfectly shaped (because they themselves are homeostatic agents in their own space). Thus, instances of communication and control between agents (at the same or different levels) are mappings between different spaces. This suggests that both evolution’s, and engineers’, hard work is to optimize the appropriate functional mapping toward robustness and adaptive function.

Next, we consider a practical example of the application of this framework to an unconventional example of cognition and flexible problem-solving: morphogenesis, which naturally leads to specific hypotheses of the origin of larger biological Selves (scaling) and its testable empirical (biomedical) predictions (Dukas, 1998). This is followed with an exploration of the implications of these concepts for evolution, and a few remarks on consciousness.

## Somatic Cognition: An Example of Unconventional Agency in Detail

“Again and again terms have been used which point not to physical but to psychical analogies. It was meant to be more than a poetical metaphor…”

– Spemann (1967)

**FIGURE 6:**
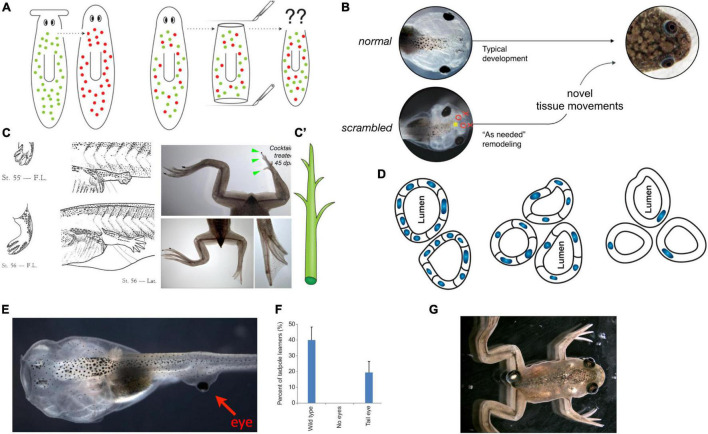
Morphogenesis as an example of collective intelligence and plasticity. The results of complex morphogenesis are the behavior in morphospace of a collective intelligence of cells. It is essential to understand this collective intelligence because by themselves, progress in molecular genetics is insufficient. For example, despite genomic information and much pathway data on the behavior of stem cells in planarian regeneration, there are no models predicting what happens when cells from a flat-headed species are injected into a round-headed species (A): what kind of head will they make, and will regeneration/remodeling ever stop, since the target morphology can never match what either set of cells expects? Development has the ability to overcome unpredictable perturbations to reach its goals in morphospace: tadpoles made with scrambled positions of craniofacial organs can make normal frogs (B) because the tissues will move from their abnormal starting positions in novel ways until a correct frog face is achieved (Vandenberg et al., 2012). This illustrates that the genetics seeds the development of hardware executing not an invariant set of movements but rather an error minimization (homeostatic) loop with reference to a stored anatomical setpoint (target morphology). The paths through morphospace are not unique, illustrated by the fact that when frog legs are induced to regenerate (C), the intermediate stages are not like the developmental path of limb development (forming a paddle and using programmed cell death to separate the digits) but rather like a plant (C′), in which a central core gives rise to digits growing as offshoots (green arrowheads) which nevertheless ends up being a very normal-looking frog leg (Tseng and Levin, 2013). (D) The plasticity extends across levels: when newt cells are made very large by induced polyploidy, they not only adjust the number of cells that work together to build kidney tubules with correct lumen diameter, but can call up a completely different molecular mechanism (cytoskeletal bending instead of cell:cell communication) to make a tubule consisting in cross-section of just 1 cell wrapped around itself; this illustrates intelligence of the collective, as it creatively deploys diverse lower-level modules to solve novel problems. The plasticity is not only structural but functional: when tadpoles are created to (E) have eyes on their tails (instead of in their heads), the animals can see very well (Blackiston and Levin, 2013), as revealed by their performance in visual learning paradigms (F). Such eyes are also competent modules: they first form correctly despite their aberrant neighbors (muscle, instead of brain), then put out optic nerves which they connect to the nearby spinal cord, and later they ignore the programmed cell death of the tail, riding it backward to end up on the posterior of the frog (G). All of this reveals the remarkable multi-scale competency of the system which can adapt to novel configurations on the fly, not requiring evolutionary timescales for adaptive functionality (and providing important buffering for mutations that make changes whose disruptive consequences are hidden from selection by the ability of modules to get their job done despite changes in their environment). Panels (A,C’,D) are courtesy of Peregrine Creative. Panel (C) is from Xenbase and Aisun Tseng. Panels (E–G) are courtesy of Douglas Blackiston. Panel (B) is used with permission from Vandenberg et al. (2012), and courtesy of Erin Switzer.

An example of TAME applied to basal cognition in an unconventional substrate is that of morphogenesis, in which the mechanisms of cognitive binding between subunits are now partially known, and testable hypotheses about cognitive scaling can be formulated [explored in detail in Friston et al. (2015a) and Pezzulo and Levin (2015, 2016)]. It is uncontroversial that morphogenesis is the result of collective activity: individual cells work together to build very complex structures. Most modern biologists treat it as clockwork [with a few notable exceptions around the recent data on cell learning (di Primio et al., 2000; Brugger et al., 2002; Norman et al., 2013; Yang et al., 2014; Stockwell et al., 2015; Urrios et al., 2016; Tweedy and Insall, 2020; Tweedy et al., 2020)], preferring a purely feed-forward approach founded on the idea of complexity science and emergence. On this view, there is a privileged level of causation—that of biochemistry—and all of the outcomes are to be seen as the emergent consequences of highly parallel execution of local rules (a cellular automaton in every sense of the term). Of course, it should be noted that the forefathers of developmental biology, such as Spemann (1967), were already well-aware of the possible role of cognitive concepts in this arena and others have occasionally pointed out detailed homologies (Grossberg, 1978; Pezzulo and Levin, 2015). This becomes clearer when we step away from the typical examples seen in developmental biology textbooks and look at some phenomena that, despite the recent progress in molecular genetics, remain important knowledge gaps (Figure 6).

### Goal-Directed Activity in Morphogenesis

Morphogenesis (broadly defined) is not only a process that produces the same robust outcome from the same starting condition (development from a fertilized egg). In animals such as salamanders, cells will also *re*-build complex structures such as limbs, no matter where along the limb axis they are amputated, and *stop when it is complete*. While this regenerative capacity is not limitless, the basic observation is that the cells cooperate toward a specific, invariant endstate (the target morphology), from diverse starting conditions, and cease their activity when the correct pattern has been achieved. Thus, the cells do not merely perform a rote set of steps toward an emergent outcome, but modify their activity in a context-dependent manner to achieve a specific anatomical target morphology. In this, morphogenetic systems meet James’ test for minimal mentality: “fixed ends with varying means” (James, 1890).

For example, tadpoles turn into frogs by rearranging their craniofacial structures: the eyes, nostrils, and jaws move as needed to turn a tadpole face into a frog face (Figure 6B). Guided by the hypothesis that this was not a hardwired but an intelligent process that could reach its goal despite novel challenges, we made tadpoles in which these organs were in the wrong positions—so-called Picasso Tadpoles (Vandenberg et al., 2012). Amazingly, they tend to turn into largely normal frogs because the craniofacial organs move in novel, abnormal paths [sometimes overshooting and needing to return a bit (Pinet et al., 2019)] and stop *when they get to the correct frog face positions*. Similarly, frog legs that are artificially induced to regenerate create a correct final form but not *via* the normal developmental steps (Tseng and Levin, 2013). Students who encounter such phenomena and have not yet been inoculated with the belief that molecular biology is a privileged level of explanation (Noble, 2012) ask the obvious (and proper) question: how does it know what a correct face or leg shape is?

Examples of remodeling, regulative development (e.g., embryos that can be cut in half and produce normal monozygotic twins), and regeneration, ideally illustrate the goal-directed nature of cellular collectives. They pursue specific anatomical states that are much larger than any individual cells and solve problems in morphospace in a context-sensitive manner—any swarm of miniature robots that could do this would be called a triumph of collective intelligence in the engineering field. Guided by the TAME framework, two questions come within reach. First, how does the collective measure current state and store the information about the correct target morphology? Second, if morphogenesis is not at the clockwork level on the continuum of persuadability but perhaps at that of the thermostat, could it be possible to re-write the setpoint without rewiring the machine (i.e., in the context of a wild-type genome)?

### Pattern Memory: A Key Component of Homeostatic Loops

Deer farmers have long known of trophic memory: wounds made on a branched antler structure in 1 year, will result in ectopic tines growing *at that same location* in subsequent years, long after the original rack of antlers has fallen off (Bubenik and Pavlansky, 1965; Bubenik, 1966; Lobo et al., 2014). This process requires cells at the growth plate in the scalp to sense, and remember for months, the location of a transient damage event within a stereotypical branched structure, and reproduce it in subsequent years by over-riding the wild-type stereotypical growth patterns of cells, instead guiding them to a novel outcome. This is an example of experience-dependent, re-writable pattern memory, in which the target morphology (the setpoint for anatomical homeostasis) is re-written within standard hardware.

**FIGURE 7:**
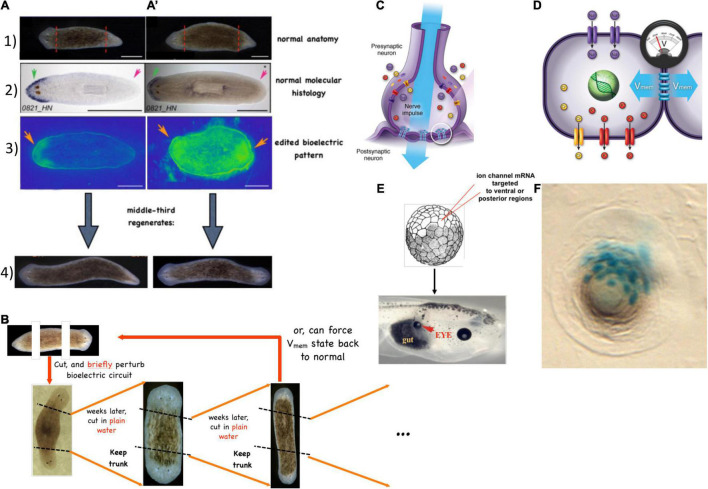
Bioelectrical pattern memories. Planarian fragments reliably regenerate whatever is missing, and stop when a correct worm is complete. Normal planaria (A) have 1 head and 1 tail (A-1), expression of anterior genes in the head (A-2), and a standing pattern of resting potential that depolarized on the end that should make a head [(A-3), revealed by voltage-reporting fluorescent dye, depolarized region marked with orange arrowhead]. When a middle portion is amputated, it regenerates to a correct 1-headed worm (A-4). It is possible to edit the information structure which encodes the target morphology (shape to which the fragments will regenerate). In worms that are anatomically normal (A′-1), with normal gene expression (A′-2), their bioelectric pattern can be altered in place [(A′-3), orange arrowheads mark the two depolarized ends] using ion channel-targeting drugs or RNAi. The result, after injury, will be a fully viable 2-headed worm (A′-4). Importantly, the pattern shown in panel (A′-3) is not a voltage map of the final 2-headed worm: it’s a map of a 1-headed animal before cutting, which already has the induced false memory indicating that a correct worm should have 2 heads. In other words, the bioelectrics can diverge from the current state—it is not simply a readout of what the anatomy is doing now, but an orthogonal information medium that is used to guide future changes of anatomy. This information is latent, only guiding the cellular collective’s anatomical homeostasis activity after injury. Thus it is also a basal example of counterfactual representation, referring to what should happen *if* an injury occurs, not what is happening now. Such changes to the bioelectric target morphology are true memories because they are re-writable but also long-term stable: if cut again, in water with no more channel-perturbing reagents, multiple rounds of regeneration of a genetically wild-type worm continue to give rise to 2-headed forms (B), which can be re-set back to normal by a different bioelectric perturbation (Oviedo et al., 2010). The control of morphology by bioelectric patterns is mediated as in the brain (C) by cells which have ion channels that set resting potential across the membrane (V_mem_) and propagate those states in computational networks to their neighbors, *via* electrical synapses known as gap junctions. All cells, not just neurons (D) do this, and bioelectric signaling is an ancient information processing modality that pre-dates neurons and brains (Fields et al., 2020; Levin, 2021a). The ability of voltage states to functionally specify modular anatomy is seen when an ion channel is used to set membrane voltage in endodermal cells fated to be gut, to an eye-like bioelectric prepattern (E), which then create an eye on the gut (red arrowhead) (Pai et al., 2012). This phenomenon has 2 levels of instruction (F): in addition to our use of voltage to instruct shape at the organ level (not micromanaging individual eye components), the ion channel mRNA-injected cells (cyan β-galactose marker) further instruct their neighbors (brown cells) to participate in forming this ectopic lens. Images in panels (C,D) are courtesy of Peregrine Creative. Images in panels (A,A′) are taken with permission from Durant et al. (2017). Embryo image in panel (E) is from Xenbase. Panel (F) is used with permission from Zahn et al. (2017).

Planarian flatworms can be cut into multiple pieces, and each fragment regenerates precisely what is missing at each location (and re-scales the remaining tissue as needed) to make a perfect little worm (Cebrià et al., 2018). Some species of planaria have an incredibly messy genome—they are mixoploid due to their method of reproduction: fission and regeneration, which propagates any mutations that don’t kill the stem cell and expands it throughout the lineage [reviewed in Fields et al. (2020)]. Despite the divergence of genomic information, the worms are champion regenerators, with near 100% fidelity of anatomical structure. Recent data have identified one set of mechanisms mediating the ability of the cells to make, for example, the correct number of heads: a standing bioelectrical distribution across the tissue, generated by ion channels and propagated by electrical synapses known as gap junctions (Figures 7A–D). Manipulation of the normal voltage pattern by targeting the gap junctions (Sordillo and Bargmann, 2021) or ion channels can give rise to planaria with one, two, or 0 heads, or heads with shape (and brain shape) resembling other extant species of planaria (Emmons-Bell et al., 2015; Sullivan et al., 2016). Remarkably, the worms with abnormal head number are *permanently* altered to this pattern, despite their wild-type genetics: cut into pieces with no further manipulations, the pieces continue to regenerate with abnormal head number (Oviedo et al., 2010; Durant et al., 2017). Thus, much like the optogenetic techniques used to incept false behavioral memories into brains (Vetere et al., 2019), modulation of transient bioelectric state is a conserved mechanism by which false pattern memories can be re-written into the genetically-specified electrical circuits of a living animal.

### Multi-Scale Competency of Growth and Form

A key feature of morphogenesis is that diverse underlying molecular mechanisms can be deployed to reach the same large-scale goal. This plasticity and coarse-graining over subunits’ states is a hallmark of collective cognition, and is also well known in neuroscience (Prinz et al., 2004; Otopalik et al., 2017). Newt kidney tubules normally have a lumen of a specific size and are made up (in cross section) of 8–10 cells (Fankhauser, 1945a,b). When the cell size is experimentally enlarged, the same tubules are made of a smaller number of the bigger cells. Even more remarkable than the scaling of the cell number to unexpected size changes (on an ontogenetic, not evolutionary, timescale) is the fact that if the cells are made really huge, *just one cell* wraps around itself and still makes a proper lumen (Figure 6D). Instead of the typical cell-cell interactions that coordinate tubule formation, cytoskeletal deformations within one cell can be deployed to achieve the same end result. As in the brain, the levels of organization exhibit significant autonomy in the details of their molecular activity but are harnessed toward an invariant system-level outcome.

### Specific Parallels Between Morphogenesis and Basal Cognition

The plasticity of morphogenesis is significantly isomorphic to that of brains and behavior because the communication dynamics that scale individual neural cells into a coherent Self are ones that evolution honed long before brains appeared, in the context of morphogenic control (Fields et al., 2020), and before that, in metabolic control in bacterial biofilms (Prindle et al., 2015; Liu et al., 2017; Martinez-Corral et al., 2019; Yang et al., 2020). Each genome specifies cellular hardware that implements signaling circuits with a robust, reliable default “inborn” morphology—just as genomes give rise to brain circuits that drive instinctual behavior in species that can build nests and do other complex things with no training. However, evolution selected for hardware that can be reprogrammed by experiences, in addition to its robust default functional modes—in body structure, as well as in brain-driven behavior. Many of the brain’s special features are to be found, unsurprisingly, in other forms outside the central nervous system. For example, mirror neurons and somatotopic representation are seen in limbs’ response to injury, where the type and site of damage to one limb can be read out within 30 s from imaging the opposite, un-injured limbs (Busse et al., 2018). Table 2 shows the many parallels between morphogenetic and cognitive systems.

**TABLE 2:** isomorphism between cognition and pattern formation.

Cognitive concept	Morphogenetic concept
Patterns of activation across neural networks processing information	Differential patterns of V_mem_ across tissue formed by propagation of bioelectric states through gap junction synapses.
Local field potential (EEG)	V_mem_ distribution of cell group
Intrinsic plasticity	Change of ion channel expression based on V_mem_ levels
Synaptic plasticity	Change of cell:cell connectivity *via* V_mem_’s regulation of gap junctional connectivity
Activity-dependent transcriptional changes	Bioelectric signals’ regulating gene expression during patterning
Neuromodulation, and neurotransmitters controlled by electrical dynamics to regulate genes in neurons	Developmental (pre-nervous) signaling *via* the same neurotransmitters (e.g., serotonin) moving under control of bioelectrical gradients to regulate second messenger pathways and gene expression.
Direct transmission	Cell:cell sharing of voltage *via* nanotubes or gap junctions
Volume transmission	Cell:cell communication *via* ion levels outside the membrane or voltage-dependent neurotransmitter release
Synaptic Vesicles	Exosomes
Sensitization	Cells become sensitized stimuli, such as for example to BMP antagonists during development
Functional lateralization	Left-right asymmetry of body organs
Taste and olfactory perception	Morphogenetic signaling by diffusible biochemical ligands
Activity-dependent modification of CNS	Control of anatomy by bioelectric signaling within those same cells
Critical plasticity periods	Competency windows for developmental induction events
Inborn behaviors (instincts)	Emergent morphogenetic cascades as “default” outcomes of a genetically-specified bioelectric hardware—hardwired patterning programs (mosaic development
Voluntary movement	Remodeling, regeneration, metamorphosis
Memory	Short range: epigenetic cell memory Medium range: Regeneration of specific body organs. Long range: Morphological homeostasis over decades as individual cells senesce; altering basic body anatomy in planaria by direct manipulation of bioelectric circuit
Counterfactual memories	Ability of 1-headed planarian bodies to store bioelectric patterns indicative of 1-headed or 2-headed forms, which are latent memories that become instructive upon damage to the organism.
Perceptual Bistability	Cryptic Planaria, induced by gap-junctional disruption, fragments of which stochastically regenerate as 1-headed or 2-headed forms, shifting between two different bioelectrical representations of a target morphology (pattern memory).
Edge detection in retina	Sharp boundaries between regions of different V_mem_ induce downstream gene expression and morphogenetic outcomes
Pattern completion ability of neural networks (e.g., attractor nets)	Regeneration of missing parts in partial fragments (e.g., planaria, salamander appendages, etc.)
Forgetting	Degradation of target morphology setpoint information leading to cancer and loss of regenerative ability
Addiction	Dependency on cellular signals, such as nerve addiction in limb regeneration and cancer addiction to specific molecules.
Encoding	Representation of patterning goal states by bioelectric properties of tissue
Visual system feature detection	Organ-level monitoring of body configuration and detection of specific boundaries by tissue (such as the V_mem_ boundary that drives brain morphogenesis)
Holographic (distributed) storage	Any small piece of a planarian remembers the correct pattern (even if it has been re-written)
Behavioral plasticity	Regulative developmental programs and regenerative capacity
Self-modeling	Representations of current and future morphogenetic states by bioelectric patterns such as the planarian prepattern or the bioelectric face pattern in vertebrates
Goal-seeking	Embryogenesis and regeneration work toward a specific target configuration despite perturbations
Adaptivity and Intelligence	Morphological rearrangements carry out novel, not hardwired, movements to reach the same anatomical configuration despite unpredictable initial starting state
Age-dependent cognitive decline	Age-dependent loss of regenerative ability
Top-down control	Place conditioning for drug effects—top-down control of signaling pathways

*Possible mapping of concepts in cognitive neuroscience to examples in pattern formation.*

### Not Just Philosophy: Why These Parallels Matter

The view of anatomical homeostasis as a collective intelligence is not a neutral philosophical viewpoint—it makes strong predictions, some of which have already borne fruit. It led to the discovery of reprogrammable head number in planaria (Nogi and Levin, 2005) and of pre-neural roles for serotonin (Fukumoto et al., 2005a,b). It explains the teratogenicity for pre-neural exposure to ion channel or neurotransmitter drugs (Hernandez-Diaz and Levin, 2014), the patterning defects observed in human channelopathies in addition to the neurological phenotypes [reviewed in Srivastava et al. (2020)], and the utility of gap junction blockers as general anesthetics.

Prediction derived from the conservation and scaling hypotheses of TAME can be tested *via* bioinformatics. Significant and specific overlap are predicted for genes involved in morphogenesis and cognition (categories of memory and learning). This is already known for ion channels, connexin (gap junction) genes, and neurotransmitter machinery, but TAME predicts a widespread re-use of the same molecular machinery. Cell-cell communication and cellular stress pathways should be involved in internal conflict between psychological modules (Reinders et al., 2019) and social behavior, while memory genes should be identified in genetic investigations of cancer, regeneration, and embryogenesis.

Another key prediction that remains to be tested (ongoing in our lab) is trainability of morphogenesis. The collective intelligence of tissues could be sophisticated enough to be trainable *via* reinforcement learning for specific morphological outcomes. Learning has been suggested by clinical data in the heart (Zoghi, 2004), bone (Turner et al., 2002; Spencer and Genever, 2003), and pancreas (Goel and Mehta, 2013). It is predicted that using rewards and punishments (with nutrients/endorphins and shock), not micromanagement of pathway hardware, could be a path to anatomical control in clinical settings, whether for morphology or for gene expression (Biswas et al., 2021). This would have massive implications for regenerative medicine, because the complexity barrier prevents advances such as genomic editing from impacting e.g., limb regeneration in the foreseeable future. The same reasons for which we would train a rat for a specific behavior, rather than control all of the relevant neurons to force it to do it like a puppet, explain why the direct control of molecular hardware is a far more difficult biomedical path than understanding the sets of stimuli that could motivate tissues to build a specific desired structure.

The key lesson of computer science has been that even with hardware we understand (if we built it ourselves), it is much more efficient and powerful to understand the software and evince desired outcomes by the appropriate stimulation and signaling, not physical rewiring. If the hardware is reprogrammable (and it is here argued that much of the biological hardware meets this transition), one can offload much of the complexity onto the system itself, taking advantage of whatever competence the sub-modules have. Indeed, neuroscience itself may benefit from cracking a simpler version of the problem, in the sense of neural decoding, done first in non-neural tissues.

### Non-neural Bioelectricity: What Bodies Think About

The hardware of the brain consists of ion channels which set the cells’ electrical state, and controllable synapses (e.g., gap junctions) which can propagate those states across the network. This machinery, including the neurotransmitters that eventually transduce these computations into transcriptional and other cell behaviors, is in fact highly conserved and present in all cells, from the time of fertilization (Figures 7C,D). A major difference between neural and non-neural bioelectricity is the time constant with which it acts [brains speed up the system into millisecond scales, while developmental voltage changes occur in minutes or hours (Harris, 2021; Levin, 2021a)]. Key aspects of this system in any tissue that enable it to support flexible software include the fact that both ion channels and gap junctions are themselves voltage sensitive—in effect, they are transistors (voltage-gated current conductances). This enables evolution to exploit the laws of physics to rapidly generate very complex circuits with positive (memory) and negative (robustness) feedback (Law and Levin, 2015; Cervera et al., 2018, 2019a,b,2020a). The fact that a transient voltage state passing through a cell can set off a cycle of progressive depolarization (like an action potential) or gap junctional (GJ) closure means that such circuits readily form dynamical systems memories which can store different information and change their computational behavior without changing the hardware (i.e., not requiring new channels or gap junctions) (Pietak and Levin, 2017); this is obvious in the action potential propagations in neural networks but is rarely thought about in development. It should be noted that there are many additional biophysical modalities, such as parahormones, volume conduction, biomechanics (strain and other forces), cytoskeletal dynamics, and perhaps even quantum coherence events that could likewise play interesting roles. These are not discussed here only due to length limitations; instead, we are focusing on the bioelectric mechanisms as one particularly illustrative example of how evolution exploits physics for computation and cognition.

Consistent with its proposed role, slowly-changing resting potentials serve as instructive patterns guiding embryogenesis, regeneration, and cancer suppression (Bates, 2015; Levin et al., 2017; McLaughlin and Levin, 2018). In addition to the pattern memories encoded electrically in planaria (discussed above), bioelectric prepatterns have also been shown to dictate the morphogenesis of the face, limbs, and brain, and function in determining primary body axes, size, and organ identity [reviewed in Levin and Martyniuk (2018)]. One of the most interesting aspects of developmental bioelectricity is its modular nature: very simple voltage states trigger complex, downstream patterning cascades. As in the brain, modularity goes hand-in-hand with pattern completion: the ability of such networks to provide entire behaviors from partial inputs. For example, Figure 7F shows how a few cells transduced with an ion channel that sets them into a “make the eye here” trigger recruit their neighbors, in any region of the body, to fulfill the purpose of the subroutine call and create an eye. Such modularity makes it very easy for evolution to develop novel patterns by re-using powerful triggers. Moreover, as do brains, tissues use bioelectric circuits to implement pattern memories that set the target morphology for anatomical homeostasis (as seen in the planarian examples above). This reveals the non-neural material substrate that stores the information in cellular collectives, which is a distributed, dynamic, re-writable form of storage that parallels recent discoveries of how group knowledge is stored in larger-scale agents such as animal swarms (Thierry et al., 1995; Couzin et al., 2002). Finally, bioelectric domains (Pai et al., 2017, 2018; Pitcairn et al., 2017; McNamara et al., 2019, 2020) set the borders for groups of cells that are going to complete a specific morphogenetic outcome—a system-level process like “make an eye.” They define the spatio-temporal borders of the modular activity, and suggest a powerful model for how Selves scale in general.

### A Bioelectric Model of the Scaling of the Self

**FIGURE 8:**
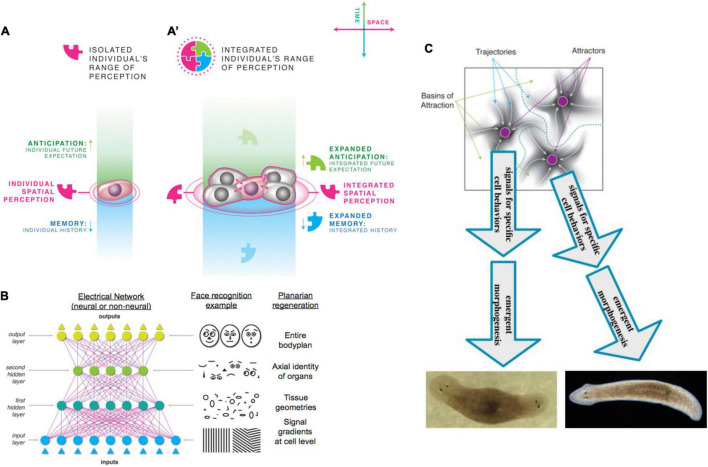
Scaling of computation in cells. Individual cells (A) have a degree of computational capacity consisting of the ability to sense local microenvironment, and some memory and ability to anticipate into the future. When assembling into networks (A′), tissues acquire the ability to sense and act at greater spatial distance, as well as gain larger capacity for memory and prediction *via* greater computational capacity. As neural networks use hidden layers to abstract patterns in data and recognize meso-scale features (B), tissue networks gain the capacity to represent information larger than the molecular and cell level: each cell’s activity (differentiation, migration, etc.) can be the result of other layers of cells processing information about current and past states, enabling decision-making with respect to tissue, organ, or whole organism-scale anatomy. (C) Much as some neural networks store individual memories as attractors in their state space, bioelectric circuits’ attractors function as pattern memories, triggering cells to execute behaviors that implement anatomical outcomes like number and location of heads in planaria. Images courtesy of Jeremy Guay of Peregrine Creative.

Gap junctional connections between cells provide an interesting case study for how the borders of the Self can expand or contract, in the case of a morphogenetic collective intelligence (Figure 8). Crucially, gap junctions [and gap junctions extended by tunneling nanotubes (Wang et al., 2010; Ariazi et al., 2017)] enable a kind of cellular parabiosis—a regulated fusion between cells that enables lateral inheritance of physiological information, which speeds up processing in the same way that lateral gene inheritance potentiates change on evolutionary timescales. The following is a case study hypothesizing one way in which evolution solves the many-into-one problem (how competent smaller Selves bind into an emergent higher Self), and how this process can break down leading to a reversal (shrinking) of the Self boundary (summarized in Table 3).

**TABLE 3:** An example of the scaling of cognition.

• Each Self has a cognitive capacity defined by the spatial, temporal, and complexity metrics on the goals it can possibly pursue. • Biological Selves scale up by cells’ joining into computational networks that can pursue larger-scale (anatomical, not just metabolic) goals. • Networks increase the spatial reach of sensing and actuation, and increase the computational capacity which allows scaling up of goals and of the states that can induce stress. • Bodies consist of components which are themselves competent (goal-seeking modules that navigate their own spaces) and can achieve specific outcomes despite perturbations and changing conditions. • Gap junctions are a unique scaling mechanism which, by linking cells’ internal milieus, wipes ownership information on signaling molecules. This partially erases the informational identity of the cellular subunits, driving up cooperation and resulting in novel tissue and organ-level Selves with morphological-scale goals. • Bioelectric networks underlie the computations of cell collectives at the tissue, organ, and organism scale, propagating stress information, state sensing, and morphogenetic instructive cues over larger areas. • Selves can dissociate (scale down), as occurs in cancer, by shrinking the computational boundaries of some subunits that de-couple from the network.

Single cells (e.g., the protozoan *Lacrymaria olor*) are very competent in handling morphological, physiological, and behavioral goals on the scale of one cell. When connected to each other *via* gap junctions, as in metazoan embryos, several things happen (much of which is familiar to neuroscientists and workers in machine learning in terms of the benefits of neural networks) that lead to the creation of a Self with a new, larger cognitive boundary. First, when cells join into an electrochemical network, they can now sense events, and act, on a much larger physical “radius of concern” than a single cell. Moreover, the network can now integrate information coming from spatially disparate regions in complex ways that result in activity in other spatial regions. Second, the network has much more computational power than any of its individual cells (nodes), providing an IQ boost for the newly formed Self. In such networks, Hebbian dynamics on the electrical synapse (GJ) can provide association between action in one location and reward in another, which enables the system to support credit assignment at the level of the larger individual.

The third consequence of GJ connectivity is the partial dissolution of informational boundaries between the subunits. GJ-mediated signals are unique because they give each cell immediate access to the internal milieu of other cells. A conventional secreted biochemical signal arrives from the outside, and when it triggers cell receptors on the surface, the cell clearly knows that this information originated externally (and can be attended to, ignored, etc.)—it is easy to maintain boundary between Self and world. However, imagine a signal like a calcium spike originating in a cell due to some damage stimulus for example. When that calcium propagates onto the GJ-coupled neighbor, there are no metadata on that signal marking its origin; the recipient cell only knows that a calcium transient occurred, and cannot tell that this information does not belong to it. The downstream effects of this second messenger event are a kind of false memory for the recipient cell, but a true memory for the collective network of the stimulus that occurred in one part of the individual. This wiping of ownership information for GJ signals as they propagate through the network is critical to enabling a partial “mind meld” between the cells: keeping identity (in terms of distinct individual history of physiological states—memory) becomes very difficult, as small informational molecules propagate and mix within the network. Thus, this property of GJ coupling promotes the creation of a larger Self by partially erasing the mnemic boundaries between the parts which might impair their ability to work toward a common goal. This is a key part of the scaling of the Self by enlarging toward common goals—not by micromanagement, but by bringing multiple subunits into the same goal-directed loop by tightly coupling the sensing, memory, and action steps in a syncytium where all activity is bound toward a system-level teleonomic process. When individual identities are blurred in favor of longer time-scale, larger computations in tissues, small-horizon (myopic) action in individual cells (e.g., cancer cells’ temporary gains followed by maladaptive death of the host) leads to a more adaptive longer-term future as a healthy organism. In effect, this builds up long-term collective rationality from the action of short-sighted irrational agents (Sasaki and Biro, 2017; Berdahl et al., 2018).

**FIGURE 9:**
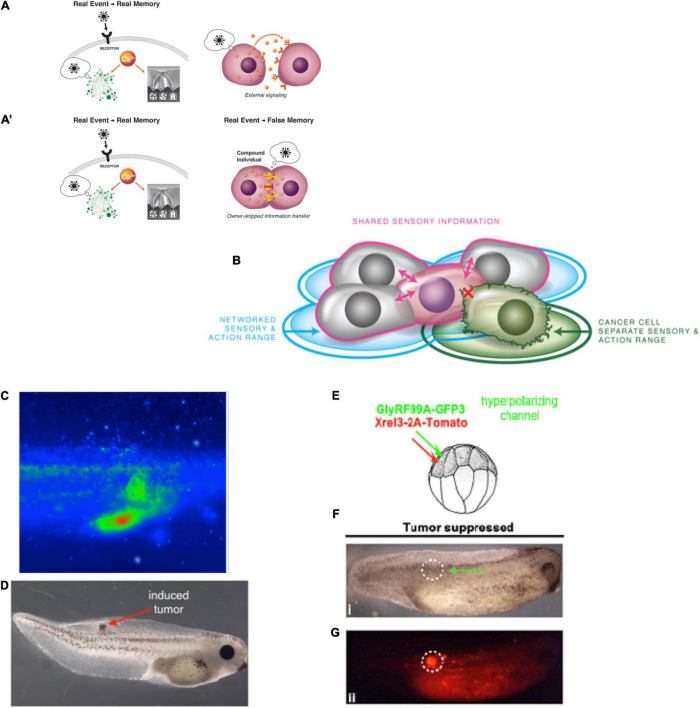
Gap junctions and the cellular collective. Communication *via* diffusible and biomechanical signals can be sensed by receptors at the membrane as messages coming from the outside of a cell (A). In contrast, cells coupled by gap junctions enable signals to pass directly from one cell’s internal milieu into another. This forms a partial syncytium which helps erase informational boundaries between cells, as memory molecules (results of pathway dynamics) propagate across such cell groups without metadata on which cell originated them. The versatile gating of GJ synapses allows the formation of multicellular Selves that own memories of physiological past events at the tissue level (not just individual cells’) and support larger target patterns, enabling them to cooperate to make complex organs (B). This process can break down: when oncogenes are expressed in tadpoles, voltage dye imaging (C) reveals the abnormal voltage state of cells that are disconnected bioelectrically from their neighbors, reverting to an ancient unicellular state (metastasis) that treats the rest of the body as external environment and grows out of control as tumors (D). This process can be prevented (Chernet and Levin, 2013a,b; Chernet et al., 2016) by artificially regulating their bioelectric state [e.g., co-injecting a hyperpolarizing channel with the oncogene, (E)]. In this case the tissue forms normally [(F), green arrow], despite the very strong presence of the oncogene [(G), red label]. This illustrates the instructive capacity of bioelectric networks to dominate single cell and genetic states to control large-scale tissue outcomes. Panels (A,A′,B) courtesy of Jeremy Guay of Peregrine Creative. Panels (C–D) are used with permission from Chernet and Levin (2013a). Panels (E–G) used with permission from Chernet and Levin (2013b).

It is important to note that part of this story has already been empirically tested in assays that reveal the shrinking as well as the expansion of the Self boundary (Figure 9). One implication of these hypotheses is that the binding process can break down. Indeed this occurs in cancer, where oncogene expression and carcinogen exposure leads to a closure of GJs (Vine and Bertram, 2002; Leithe et al., 2006). The consequence of this is transformation to cancer, where cells revert to their ancient unicellular selves (Levin, 2021b)—shrinking their computational boundaries and treating the rest of the body as external environment. The cells migrate at will and proliferate as much as they can, fulfilling their cell-level goals—metastasis [but also sometimes attempting to, poorly, reboot their multicellularity and make tumors (Egeblad et al., 2010)]. The model implies that this phenotype can be reverted by artificially managing the bioelectric connections between a cell and its neighbors. Indeed, recent data show that managing this connectivity can override default genetically-determined states, inducing metastatic melanoma in a perfectly wild-type background (Blackiston et al., 2011) or suppressing tumorigenesis induced by strong oncogenes like p53 or KRAS mutations (Chernet and Levin, 2013a,b). The focus on physiological connectivity (information dynamics)—the software—is consistent with the observed facts that genetic alterations (hardware) are not necessary to either induce or revert cancer [reviewed in Chernet and Levin (2013a)].

All these dynamics lead to a few interesting consequences. GJ-mediated communications are not merely conversations (in the way that external signaling is)—they are binding, in the sense that once a GJ is open, a cell is subject to whatever comes in from the neighbor. In the same sense, having a synapse makes one vulnerable to the state of neighbors. GJs spread (dilute) the pain of depolarization, but at the same time give a cell’s neighbors the power to change its state. Compatible with the proposal that the magnitude of a Self is the scale and complexity of states by which it can be stressed, connections by tunable, dynamic GJs greatly expand the spatial, temporal, and complexity of things that can irritate cells; complex events from far away can now percolate into a cell *via* non-linear GJ paths through the network, and enabling the drive to minimize such events now necessarily involves homeostatic activity of goal states, sensing, and activity on a much larger scale. Stress signals, propagating through such networks, incentivize other regions of the tissue to act cooperatively in response to *distant* events by harnessing their selfish drive to reduce their own stress. This facilitates the coherent, system-level response to perturbations beyond their local consequences, and gives rise to larger Selves able to react coherently to stressful departures from more complex, spatially-distributed allostatic setpoints. For example, whereas a solitary cell might be stressed (and react to) abnormal local pH, cells that are part of a transplanted salamander limb will be induced to a more grandiose activity: they will change the number of fingers they produce to be correct relative to the limb’s new position in the host’s body (Ruud, 1929), a decision that involves large-scale sensing, decision-making, and control.

A fourth consequence of the coupling is that cooperation in general is greatly enhanced. In the game theory sense, it is impossible to cheat against your neighbor if you are physiologically coupled. Any positive or negative effects of a cell’s actions toward the neighboring cell are immediately propagated back to it, in effect producing one individual in which the parts cannot “defect” against each other. This dynamic suggests an interesting twist on Prisoners’ Dilemma models in which the number of agents is not fixed, because they have the options of Cooperate, Defect, Merge, and Split (we are currently analyzing such models). Specifically, merging with another agent creates an important dimensionality reduction (because defection is no longer an option); this not only changes the calculus of game theory as applied to biological interactions, but also the action space itself. These dynamics take place on a developmental timescale, complementing the rich existing literature on game theory in evolution (Maynard Smith and Szathmáry, 1995; Maynard Smith, 1999; McEvoy, 2009; Pacheco et al., 2014).

**FIGURE 10:**
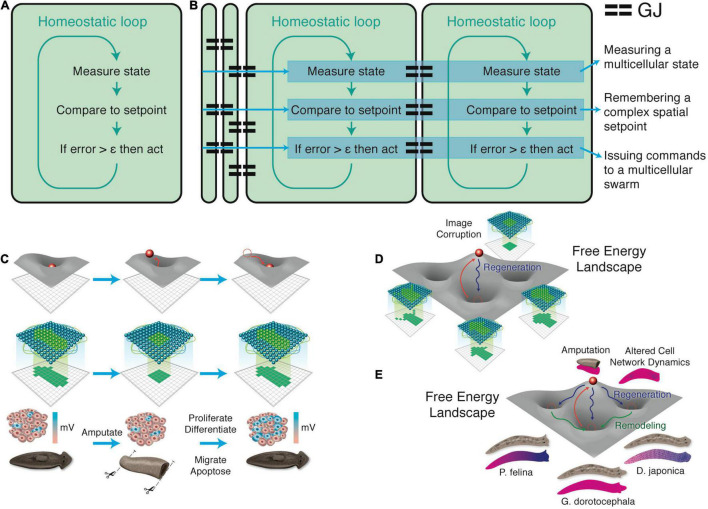
Gap junctions scale homeostatic goals. Gap junctions are a type of connection architecture that facilitates scaling of goal states (and thus expands the cognitive cone of cellular agents). A single cell’s homeostatic cycle has 3 parts: measurements, comparison to a stored setpoint, and acting *via* effectors to stay in or reach the correct region of state space (A). When coupled into gap junctions (B), each of these 3 components expands in size and complexity: the cell group is able to (1) measure a larger region (reacting to spatially more complex inputs, not just local conditions), (2) store a more complex setpoint pattern, and (3) act (deform, grow, etc.) at a scale that produces large-scale anatomical change. The goals of such networks readily map on to regeneration and regulative development (C): dynamical systems pictures of artificial neural networks as they perform pattern completion based on partial input illustrate an energy landscape with wells corresponding to stable target morphology memories. The process of completing a correct planarian pattern from a simple fragment can be modeled in this way, perhaps with overall stress levels instantiating the free energy that the system is trying to minimize (Kuchling et al., 2020b). Such attractors correspond to different possible morphologies, and indeed the normally robust regeneration toward a single pattern (D) can be modified in planaria by temporarily disrupting their gap junctional network, which causes genetically un-modified worms to nevertheless build heads appropriate to other species’ attractors in morphospace (E) (Emmons-Bell et al., 2015; Sullivan et al., 2016). Images in panels (A,B) courtesy of Jeremy Guay of Peregrine Creative. Images in panels (C–E) courtesy of Alexis Pietak.

Indeed, the smaller and larger agents’ traversal of their various spaces provides a way to think about how smaller agents’ (cell-level) simple homeostatic loops can scale up into large, organ-level anatomical homeostatic loops. Prentner recently showed how agents build up spatial models of their worlds by taking actions that nullify changes in their experience (Prentner, 2019). Working to nullify changes to one’s state that would otherwise be induced by the vagaries of external environment (and other agents) is the core of homeostasis—the action loops that seek to preserve important states against intervention and entropy. This is not only for physical movement (which results in a creature perceiving itself to be situated in spacetime) but also for other states in which actuation takes place *via* turning on/off specific genes, remodeling an anatomy, or opening/closing ion channels to change physiological state. An agent can notice patterns in what actions it had to take to keep in homeostasis despite various perturbations that occur, and based on that refine an internal model of some space within which it is acting. This is closely related to the surprise minimization framework (Friston, 2013; Friston et al., 2013; Friston K. et al., 2014), and suggests a straightforward sense in which larger Selves scale up to models of their world and themselves from evolutionary primitives such as metabolic homeostasis. Bioelectricity provides examples where cell-level physiological homeostats form networks that implement much larger-scale pattern memories as attractors, akin to Hopfield networks (Figure 10; Hopfield, 1982; Inoue, 2008; Pietak and Levin, 2017; Cervera et al., 2018, 2019a,b,2020a). This enables all tissues to participate in the kind of pattern completion seen in neural networks—a critical capability for regenerative and developmental repair (anatomical homeostasis).

With these pieces in place, it is now possible to mechanistically visualize one specific aspect of the progressive scaling that expands the cognitive light cone. Cells with a chemical receptor can engage in predictive coding to manage their sensory experience (Friston K. et al., 2014; Friston K. J. et al., 2014; Thornton, 2017). Similarly, individual cells homeostatically maintain V_mem_ (cell membrane resting potential voltage) levels. However, cells can electrically couple *via* gap junctions to create bioelectric networks that work as a kind of virtual governor—coupled oscillators possess emergent dynamics that now maintain large, spatial *patterns* of V_mem_ against perturbation with greater stability (Pietak and Levin, 2017, 2018; Cervera et al., 2018, 2019a,b,2020a,b; Pai et al., 2018; Manicka and Levin, 2019a). These spatial patterns serve as instructive pattern memories guiding the activity of a cell collective through anatomical morphospace toward the correct target morphology (Sullivan et al., 2016; Levin, 2021a; Pezzulo et al., 2021).

Voltage is especially interesting because each V_mem_ level in a single cell is a coarse-grained parameter, subsuming many distinct combinations of sodium, potassium, chloride, etc., levels, and many distinct open/closed states of particular ion channel proteins, which all result in the same resting potential. This can be seen as the minimal version of generalization—cells learning to respond to *classes* of events by transducing not specific ion levels or channel protein activity states but the macrovariable “voltage.” This in turn enables them to repurpose existing responses for novel combinations of stimuli (e.g., familiar depolarization events caused by novel ion dynamics).

Moreover, gap junctions propagate voltage states across tissue, allowing cells to respond to events that are not local in nature (larger-scale) and to respond *en masse*. More generally, this means that the input to any group of cells is produced by the output of groups of cells—sub-networks, which can be complex and highly processed over time (not instantaneous), enabling predictive coding to manage complex states (at a distance in space and time) and not only purely local, immediate sensory data. It also means that the system is extremely modular, readily connecting diverse upstream and downstream events to each other *via* the universal adapter of bioelectric states. When this is applied to the homeostatic TOTE (test-operate-exit) loop, allowing its measurement, comparison, and action modules to be independently scaled up (across space, time, and complexity metrics), this inherently expands the cognitive light cone of a homeostatic agent to enable progressively more grandiose goals.

Crucially, all of the above-mentioned aspects of the role of generic bioelectric networks underlying the scaling of Selves are not only the products of the evolutionary process, but have many functional implications for evolution itself (forming a positive feedback loop in which rising multiscale agency potentiates the evolution of increasingly more complex versions).

## Evolutionary Aspects

Developmental bioelectricity works alongside other modalities such as gene-regulatory networks, biomechanics, and biochemical systems. The TAME framework emphasizes that what makes it special is that it’s not just another micro-mechanism that developmental biologists need to track. First, developmental bioelectrics is a unique computational layer that provides a tractable entrypoint into the informational architecture and content of the collective intelligence of morphogenesis. Second, bioelectric circuits show examples of modularity, memory, spatial integration, and generalization (abstraction over ion channel microstates)—critical aspects of understanding basal origins of cognition. Developmental bioelectricity provides a bridge between the early problem-solving of body anatomy and the more recent complexity of behavioral sophistication *via* brains. This unification of two disciplines suggests a number of hypotheses about the evolutionary path that pivoted morphogenetic control mechanisms into the cognitive capacities of behavior, and thus sheds light on how Selves arise and expand.

### Somatic Bioelectrics Reveals the Origin of Complex Cognitive Systems

Developmental bioelectrics is an ancient precursor to nervous systems. Analog bioelectrical dynamics generate patterns in homogenous cell sheets and coordinate information that regulates transcription and cell behaviors. Evolution first exploited this to enable cell groups to position the body configuration in developmental morphospace, long before some cells specialized to use very fast, digital spiking as neural networks for control of behavior as movement in 3-dimensional space (Fields et al., 2020). The function of nervous systems as spatial organizers operating on data from the external world (Keijzer et al., 2013) is an adaptation built upon the prior activity of bioelectric circuits in organizing the internal morphology by processing data from the internal milieu. While neural tissues electrically encode spatial information to guide movement (e.g., memory of a maze in a rat brain) by controlling muscles, bioelectric prepatterns guide the behaviors of other cell types, on slower timescales, during development, regeneration, and remodeling toward invariant, robust anatomical configurations.

Developmental bioelectricity illustrates clearly the continuous nature of properties thought to be important for cognition, and the lack of a clean line separating brainy creatures from others. On a single-cell level, even defining a “neuron” is not trivial, as most cells possess the bioelectrical machinery and a large percentage of neuronal genes are also expressed in non-neural cells (Bucher and Anderson, 2015), while neural molecular components are found in cytonemes (Huang et al., 2019). Many channel families were likely already present in the most recent unicellular ancestor (Liebeskind et al., 2015). The phylogeny of ion channels is ancient, and the appearance of context-sensitive channels (enabling new kinds of bioelectrical feedback loops) tracks well with the appearance of complex body plans at the emergence of metazoa (Moran et al., 2015), revealing the remarkable evolutionary continuum that leads from membrane excitability in single cells to cognitive functions in advanced organisms, by way of somatic pattern control (Cook et al., 2014).

Fascinating work on bacteria has shown that prokaryotes also utilize bioelectric state for proliferation control (Stratford et al., 2019); and, paralleling the developmental data discussed above, bioelectric phenomena in bacteria scale easily from single-cell properties (Kralj et al., 2011) to the emergence of proto-bodies as bacterial biofilms. Bacterial communities use brain-like bioelectric dynamics to organize tissue-level distribution of metabolites and second messenger molecules, illustrating many of the phenomena observed in complex morphogenetic contexts, such as encoding stable information in membrane potential patterns, bistability, and spatial integration (Humphries et al., 2017; Liu et al., 2017; Larkin et al., 2018; Martinez-Corral et al., 2018; Yang et al., 2020). Not only animal lineages, but plants (Baluska and Mancuso, 2012; Volkov et al., 2019; Serre et al., 2021) use bioelectricity, as evolution frequently exploits the fact that bioelectric dynamics are a powerful and convenient medium for the computations needed to solve problems in a variety of spaces not limited to movement in 3D space.

Developmental bioelectricity helps explain how free-living cells scaled their cell-level homeostatic pathways to whole body-level anatomical homeostasis (Levin, 2019). It has long been appreciated that evolvability is potentiated by modularity—the ability to trigger complex morphogenetic cascades by a simple “master” trigger that can be re-deployed in various contexts in the body (von Dassow and Munro, 1999). Recent advances reveal that bioelectric states can form very powerful master inducers that initiate self-limiting organogenesis. For example, the action of a single ion channel can induce an eye-specific bioelectric state that creates complete eyes in gut endoderm, spinal cord, and posterior tissues (Pai et al., 2012)—locations where genetic “master regulators” like the *Pax6* transcription factor are insufficient in vertebrates (Chow et al., 1999). Likewise, misexpression of a proton pump (or a 1-h ionophore soak) to trigger bioelectric changes in an amputation wound can induce an entire 8-day cascade of building a complete tadpole tail (Adams et al., 2007; Tseng et al., 2010). This is control at the level of organ, not single cells’ fate specification, and does not require the experimenter to provide all of the information needed to build the complex appendage. Thus, bioelectric states serve as effective master regulators that evolution can exploit to make modular, large-scale changes in anatomy.

Moreover, because the same V_mem_ dynamics can be produced by many different ion channel combinations, and because bioelectric states propagate their influence across tissue distance during morphogenesis (Chernet and Levin, 2014; Pai et al., 2020), evolution is free to swap out channels and explore the bioelectrical state space: simple mutations in electrogenic genes can exert very long-range, highly coordinated changes in anatomy. Indeed, the KCNH8 ion channel and a connexin were identified in the transcriptomic analysis of the evolutionary shift between two functionally different morphologies of fin structures in fish (Kang et al., 2015). The evolutionary significance of bioelectric controls can also be seen across lineages, as some viruses evolved to carry ion channel and gap junction (Vinnexin) genes that enable them to hijack bioelectric machinery used by their target cells (Shimbo et al., 1996; Hover et al., 2017).

The unique computational capabilities of bioelectric circuits likely enabled the evolution of nervous systems, as specialized adaptations of the ancient ability of all cell networks to process electrical information as pre-neural networks (Keijzer, 2015; Fields et al., 2020). A full understanding of nervous system function must involve not only its genetics and molecular biology but also the higher levels of organization comprising dynamic physiology and computations involved in memory, decision-making, and spatio-temporal integration. The same is true for the rest of the body. For example, the realization that epithelia are the generators of bioelectric information (Robinson and Messerli, 1996) suggests models in which they act like a retina wrapped around a whole embryo (and individual organs) to preprocess electrical signals into larger-scale features and compute contrast information for downstream processing (Grossberg, 1978). The investigation of somatic bioelectric states as primitive “pattern memories” and the expansion of computational science beyond neurons will enrich the understanding of cell biology at multiple scales beyond molecular mechanisms, as is currently only done with respect to the brain (Marr, 1982). Generalizing the deep concepts of multiscale neuroscience beyond neurons (Grossberg, 1978; Pezzulo and Levin, 2015; Manicka and Levin, 2019b) is necessary for a better understanding of the tissue-level decision-making that drives adaptive development and regeneration. Conversely, advances in understanding information processing in a relatively simpler anatomical context will feed back to enrich important questions in brain science, shedding light on fundamental mechanisms by which information-processing agents (cells) work collectively to accomplish unified, complex system-level outcomes. The multi-disciplinary opportunity here is not only to gain insight into the phylogeny of nervous systems and behavior, but to *erase the artificial boundaries between scientific disciplines that focus on neurons vs. the rest of the body, with the direct consequence that a more inclusive, gradualist picture emerges of the mechanisms commonly associated with cognitive Selves*.

Ion channels and gap junctions are the hardware interface to the bioelectric computational layer within living systems. Like a retina for a brain, or a keyboard for a computer, they allow transient signals to serve as inputs to memory and decision-making networks. For any given agent (cell, tissue, etc.), its bioelectrical interface is accessed by a number of potential users. First are the neighboring agents, such as other tissues, which pass on their bioelectric state during cooperative and competitive interactions in morphogenesis. There are also commensal and parasitic microbes, which have evolved to hijack such control systems to manipulate the anatomy of the host—like the naïve bacteria on planaria that can determine head number and visual system structure in flatworm regeneration (Williams et al., 2020). Moreover, the development of pharmacological, genetic, and optogenetic tools now allows human bioengineers to access bioelectrical circuits for the control of growth and form in regenerative medicine and synthetic bioengineering contexts (Adams et al., 2013, 2014, 2016; Chernet et al., 2016; McNamara et al., 2016; Bonzanni et al., 2020). All of these manipulations can serve as catalysts, enabling an evolutionary lineage to more easily travel to regions of option space that might otherwise be separated by an energy barrier that is difficult for standard evolution to reach. In this sense, cognitive properties of developmental mechanisms help us to understand problem-solving on phylogenetic, not just ontogenetic, timescales. We next look at specific ways in which the architecture of multiscale autonomy, especially as implemented by bioelectric network mechanisms, potentiates evolution.

### Multi-Scale Autonomy Potentiates the Speed of Evolution

Deterministic chaos and complexity theory have made it very clear why bottom-up control of even simple systems (e.g., 3-body problem) can be practically impossible. This inverse problem (Lobo et al., 2014)—what control signals would induce the desired change—is not only a problem for human engineers but also for adjacent biological systems such as the microbiome or a fungus that seeks to control the behavior of an ant (Hughes et al., 2016), and most of all, for other parts of a complex system (to help control itself). Evolution tackles this task by exploiting a multiscale competency architecture (MCA), where subunits making up each level of organization are themselves homeostatic agents. It’s built on an extremely powerful design principle: error correction (Fields and Levin, 2017; Frank, 2019a,b).

The key aspect of biological modularity is not simply that complex subroutines can be triggered by simple signals, making it easy to recombine modules in novel ways (Schlosser and Wagner, 2004; Gerhart and Kirschner, 2007), but that these modules are also themselves sub-agents exhibiting infotaxis and socialtaxis, and solving problems in their own spaces (Vergassola et al., 2007; Gottlieb et al., 2013; Karpas et al., 2017). When an eye primordium appears in the wrong place (e.g., a tadpole tail), it still forms a correctly patterned, functional organ, manages to get its data to the brain (*via* spinal cord connection) to enable vision (Figure 6E), and (if somewhere in the head) moves to the correct place during metamorphosis (Vandenberg et al., 2012). When cells are artificially made to be very large and have several times the normal genetic material, morphogenesis adapts to this and still builds an overall correct animal (Fankhauser, 1945a,b). These are goal-directed (in the cybernetic sense) processes because the system can reach a specific target morphology (and functionality) state despite perturbations or changes in local/starting conditions or the basic underlying components. Regeneration is the most familiar example of this, but is just a special case of the broader phenomenon of anatomical homeostasis. Homeostatic loops operating over large-scale anatomical states have several (closely related) key implications for the power and speed of evolution.

First, it greatly smoothes the fitness landscape. Consider two types of organisms: one whose subsystems mechanically follow a hardwired (genetically-specified) set of steps (A, passive, or merely structural modularity), and one whose modules optimize a reward function (B, multi-scale competency of modules). Mutations that would be detrimental in A (e.g., because they move the eye out of its optimal position) are neutral in B, because the competency of the morphogenetic subsystems repositions the eye even if it starts out somewhere else. Thus, MCA shields from selection some aspects of mutations’ negative effects (which inevitably are the bulk of random mutations’ consequences). The primary reason for the anatomical homeostasis seen in regulative development and regeneration may be for dealing, not with damage, but with deviations from target morphology induced by mutations. This is certainly true at the scale of tissues during the lifetime of an individual [as in the inverse relationship between regeneration and cancerous defection from large-scale target morphology (Levin, 2021b)], but may be true on evolutionary time scales as well.

Second, MCA reduces apparent pleiotropy—the fact that most mutations have multiple effects (Boyle et al., 2017). For example, a change in an important canonical signaling pathway such as Wnt or BMP (Raible and Ragland, 2005) is going to have numerous consequences for an organism. Suppose a mutation appears that moves the mouth off of its optimal position (bad for fitness) but also has some positive (adaptive) feature elsewhere in the body. In creatures of type A, the positive aspects of that mutation would never be seen by selection because the malfunctioning mouth would reduce the overall fitness or kill the individual outright. However, in creatures of type B, the mouth could move to its optimal spot (Vandenberg et al., 2012), enabling selection to independently evaluate the other consequence of that mutation. Creatures possessing MCA could reap the benefit of positive consequences of a mutation while masking its other effects *via* local adjustments to new changes that reduce the penalties (an important kind of buffering). In effect, evolution doesn’t have to solve the very difficult search problem of “how to improve feature X without touching features Y. Z which already work well,” and reaps massive efficiency (time) savings by not having to wait until the search process stumbles onto a way to directly encode an improvement that is either isolated from other features, or improves them all simultaneously (Wagner et al., 2007; Melo et al., 2016).

Third, MCA allows systems not only to solve problems, but also to exploit opportunities. A lineage has the chance to find out what pro-adaptive things a mutation can do, because competency hides the negative consequences. This gives time for new mutations to appear that hardwire the compensatory changes that had to be applied—an analogy to the proposed Baldwin effect (Hogenson, 2001; Downing, 2004; Robinson and Barron, 2017). This enables the opportunity to exploit the possibility space more freely, providing a kind of patience or reduction of the constraint that evolutionary benefits have to be immediate in order to propagate—it effectively reduces the short-sightedness of the evolutionary process. Indeed, multiscale competency is beneficial not only for natural evolution, but also for soft robotics and synthetic bioengineering because it helps cross the sim-to-real gap: models do not have to be 100% realistic to be predictively useful if the component modules can adaptively make up for some degree of deficiency in the controller design (Brooks, 1986).

Fourth, the homeostatic setpoint-seeking architecture makes the relationship between genotype and anatomical phenotype more linear (Muller and Schuppert, 2011; Lobo et al., 2014), improving controllability (Liu et al., 2011; Gao et al., 2014; Posfai et al., 2016). By using a top-down control layer to encode the patterns to which competent subunits operate, living systems do not need to solve the difficult inverse problems of what signals to send their subsystems to achieve high-level outcomes. Bioelectric pattern memories (such as the voltage distribution that tells wild-type planarian cells whether to build 1 head or 2) exploit a separation of data from the machine itself, which makes it much easier to make changes. Evolution does not need to find changes at the micro level but can also simply change the information encoded in the setpoints, such as the electric face prepattern (Vandenberg et al., 2012), which allows it to re-use the same exact implementation machinery to build something that can be quite different. The ability to rely on a non-zero IQ for your component modules (thus delegating and offloading complex regulatory chains) is an important affordance (Watson et al., 2010; Friston et al., 2012) for the evolutionary process. It means that the larger system’s evolution is in effect searching an easier, less convoluted control, signaling, or reward space—this massive dimensionality reduction offers the same advantages human engineers have with agents on the right side of the persuadability scale. It is no accident that learning in the brain, and behavioral systems, eventually exapted this same architecture and indeed the exact same bioelectrical machinery to speed up the benefits of evolution.

A significant brake on the efficiency of evolution, as on machine learning (indeed, all learning) is credit assignment: which change or action led to the improvement or reward? When a collection of cells known as a “rat” learns to press a level and get a reward, no individual cell has the experience of interacting with a lever and receiving the nutrient. What enables the associative memory in this collective intelligence are the delay lines (nervous system) between the paws and the intestinal lining which provide a kind of patience—a tolerance of the temporal delay between the action and the reward and the ability to link extremely diverse modules on both ends (different kinds of actions can be linked to arbitrary rewards). MCA does the same thing for evolutionary learning (Watson et al., 2014, 2016; Power et al., 2015; Watson and Szathmary, 2016; Kouvaris et al., 2017), making it easier for systems to reap selection rewards for arbitrary moves in genotype space. This effectively raises the IQ of the evolutionary search process. Much as (Figure 5) an agent’s sophistication can be gauged by how expertly and efficiently it navigates an arbitrary search space and its local optima, the traversal of the evolutionary search process can be made less short-sided by homeostatic activity within the physiological layer that sits between genotype and phenotype.

There is an adaptation tradeoff between robustness (e.g., morphogenesis to the same pattern despite interventions and changing conditions) and responsiveness to environment (context sensitivity), perhaps similar to the notion of criticality (Beggs, 2008; Hankey, 2015). The plasticity and goal-directedness of modules (as opposed to hardwired patterns) serve to reduce the sim-to-real gap (Kriegman et al., 2020b): because the current environment always offers novel challenges compared to prior experiences which evolution (or human design) uses to prepare responses, the MCA architecture doesn’t take history too seriously, relying on plasticity and problem-solving more than on fine-tuning micromodels of what to do in specific cases. Biology reaps the benefits of both types of strategies by implementing anatomical homeostasis that coarse-grains robustness by making stability applying to large outcomes, such as overall anatomy, not to the microdetails of cell states. The scaling of homeostatic loops makes it possible to achieve both: consistent results and environmental sensitivity. These dynamics apply in various degrees to the numerous nested, adjacent, and overlapping sub-agents that make up any biological system. Cooperation results not from altruistic actions between Selves, but by the expansion of the borders of a single Self *via* scaling of the homeostatic loops. On this view, cancer cells are not more selfish than tissues—they are all equally selfish, but maintain goals appropriate to smaller scales of Selves. Indeed, even the parts of one normal body don’t perfectly cooperate—this is as true in development (Gawne et al., 2020) as it is in cognitive science (Dorahy et al., 2014; Reinders et al., 2018, 2019). A picture is emerging of how evolution exploits the local competency of modules, competing and cooperating, to scale these subsystems’ sensing, actuation, and setpoint memories to give rise to coherent larger-scale Selves. Overall, the TAME framework addresses functional aspects only, and is compatible with several views on phenomenal consciousness in compound Selves (Chalmers, 1996). However, it does have a few implications for the study of Consciousness.

## Consciousness

While the TAME framework focuses on 3rd-person observable properties, it does make some commitments to ways of thinking about consciousness. Provisionally, I suggest that consciousness also comes in degrees and kinds (is not binary) for the same reasons argued for continuity of cognition: if consciousness is fundamentally embodied, the plasticity and gradual malleability of bodies suggests that it is a strong requirement for proponents of phase transitions to specify what kind of “atomic” (not further divisible) bodily change makes for a qualitative shift in capacity consciousness. Another implication of TAME is that while “embodiment” is critical for consciousness, it is not restricted to physical bodies acting in 3D space, but also includes perception-action systems working in all sorts of spaces. This implies, counter to many people’s intuitions, that systems that operate in morphogenetic, transcriptional, and other spaces should also have some (if very minimal) degree of consciousness. This in turn suggests that an agent, such as a typical modern human, is really a patchwork of many diverse consciousnesses, only one of which is usually capable of verbally reporting its states (and, not surprisingly, given its limited access and self-boundary, believes itself to be a unitary, sole owner of the body).

What is necessary for consciousness? TAME’s perspective is fundamentally that of the primacy of goal-directed activity. Thus, consciousness accompanies specific types of cognitive processes which exert energy toward goals, but as described above, those processes can take forms very divergent from our typical brain-centered view. Unlike other panpsychist views, TAME does not claim that mind is inevitably baked in regardless of physical implementation or structure. Causal structure and cybernetic properties of the embodiment are key determinants of consciousness capacity. However, as the minimal degree of internal self-determination and goal-directedness is apparently present even in particles (Feynman, 1942; Georgiev and Georgiev, 2002; Ogborn et al., 2006; Kaila and Annila, 2008; Ramstead et al., 2019; Kuchling et al., 2020a), there may be no true “0” on the scale of consciousness in the universe. While simple accretion does not magnify the nano-goal-directed activity and indeterminate action of particles (e.g., rocks are not more conscious, and probably less, than particles in specific contexts), biological organization does amplify it, resulting in scaling up of sentience.

Of course, these implications will be unpalatable conclusions for many. It should be kept in mind that TAME is compatible with several different views on consciousness, and does not need to commit to one specific philosophy. It is fundamentally a framework for enabling empirical experiments, and its practical utility remains, regardless of the above speculations. Moreover, I remain skeptical about being able to say anything definitive about consciousness *per se* (as distinct from correlates of consciousness) from a 3rd-person, objective perspective. Thus, however unappealing the above view may be, I see no way of rigorously showing why any other claim about consciousness and what it requires is demonstrably better.

An emphasis on somatic plasticity has additional practical implications, being focused on the functional splitting and joining of agents’ parts. For example, the ancient question of “where does it all come together?” in the brain, with respect to the unified character of consciousness, is one of those pseudo-problems that is dispelled by a framework like TAME that focuses on multi-scale architecture. How big should a place where it all comes together be? If it can be ∼140 mm wide, then the answer is, the whole brain. One could decide that it should be smaller (the human pineal gland is ∼7 mm wide), but then the question is, why not smaller still—given the cellular components of the pineal (or any piece of the brain) and the molecular organelles inside a pineal gland cell, one would always need to ask “but where does it all come together *inside there*?” of whatever piece of the brain is taken to be the seat of consciousness. The multi-scale nature of biology means that there is no privileged size scale for any homunculus.

Another important idea with respect to consciousness is “What is it like to be” a given agent (Nagel, 1974). Sensory augmentation, neural link technologies, and bioengineering produce tractable model systems in novel cognitive architectures, such as 2-headed planaria where the brains are connected by a central nervous system (Figure 7B), to help study the functional aspects of this cognitive re-shuffling. TAME’s focus on the fact that all cognitive architectures are inevitably composites emphasizes that the parts can be rearranged; thus, the Subject of cognition can change “on the fly,” not merely during evolutionary timescales. Thus, the basic question of philosophy of mind—what’s it like to be animal X (Nagel, 1974)—is just a first-order step on a much longer journey. The second-order question is, what’s it like to be a caterpillar, slowly *changing* into a butterfly as its brain is largely dissolved and reassembled into a different architecture for an animal whose sense organs, effectors, and perhaps overall Umwelt is completely different. All of this raises fascinating issues of first person experience not only in purely biological metamorphoses (such as human patients undergoing stem cell implants into their brains), but also technological hybrids such as brains instrumentized with novel sensory arrays, robotic bodies, software information systems, or brains functionally linked to other brains (Warwick et al., 1998; Demarse et al., 2001; Potter et al., 2003; Bakkum et al., 2007a,b; Tsuda et al., 2009; Cohen-Karni et al., 2012; Giselbrecht et al., 2013; Aaser et al., 2017; Ricotti et al., 2017; Ding et al., 2018; Mehrali et al., 2018; Anderson et al., 2020; Ando and Kanzaki, 2020; Merritt et al., 2020; Orive et al., 2020; Saha et al., 2020; Dong et al., 2021; Li et al., 2021; Pio-Lopez, 2021). The developmental approach to the emergence of consciousness on short, ontogenetic timescales complements the related question on phylogenetic timescales, and is likely to be a key component of mature theories in this field.

**FIGURE 11:**
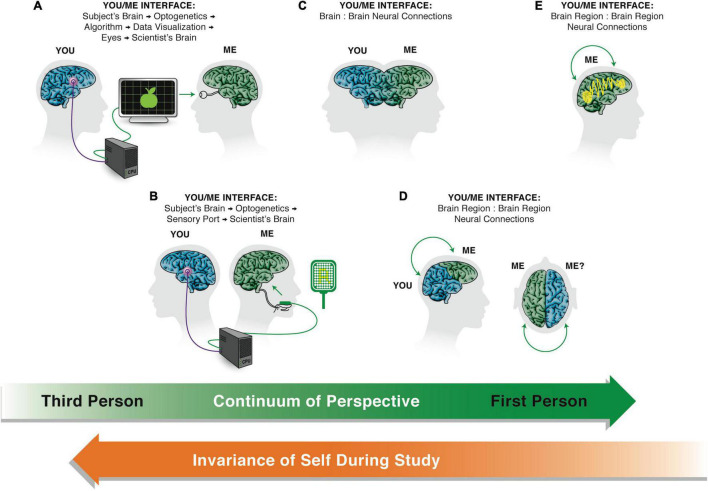
Technology reveals gradualism in Descartes’ cut. The apparent fundamental gulf between first person perspective (what is it like to *be* a specific Self) and third person perspective (external scientific study of that cognitive system) can be seen to also be a gradual continuum, when modern technology is used to expand heterophenomenology (Dennett, 1991). On the left side of the continuum (A) is a traditional 3-rd person scenario of an agent studying another by measuring its physical states: cognitive states can be inferred but not directly experienced (correlates of consciousness), giving rise to the problem of other minds, and a firm distinction between “you” and “me.” However, sensory substitution and augmentation technology now enables the plugging of various peripherals and sensors directly into the nervous system of subjects, and thus it is possible to connect the output of electrophysiology equipment directly into the subject’s brain (B). For example, if the output of a multielectrode array recording neural activity of subject #1 is connected directly to the brainport device (Danilov and Tyler, 2005) of subject #2 (e.g., a scientist), this allows #1’s mental states to more directly provide input into #2’s sensory stream. This can be made even more direct by fusing portions of two brains directly, during embryogenesis, illustrating that the strength of boundaries between “you” and “me” is variable; this configuration may seem far-fetched, but note that it can be readily produced today in animal model systems, and the only barrier to such configurations is ethical, not empirical or logical (C). It’s critical to note that these fusion experiments are not just aberrant corner cases, because all brains are *already* fusions of neural modules. Single subjects’ brains consist of two hemispheres which must communicate to give rise to a coherent, centralized perception of “me” despite being made of communicating parts, and can be dissociated by commissurotomy (D). Indeed, beyond the two hemispheres, any brain is a collective of smaller active subunits (E) that must all communicate as a collection of cells (each neuron is part of the neighboring neural cell’s “external environment”). This gradient of diverse connections, whether electronic or biological, between and within brains and brain components can be reproduced or expanded upon to whatever degree necessary by biological or technological fusion among subjects. The technological aspect of TAME is that we must develop frameworks that deal not only with standard embodiments of mind as happened to be produced by the path evolution took through life on Earth, but all logically and empirically possible configurations that could evolve, be designed, or both, whether on Earth or in exobiological contexts. The hierarchical, not monadic, structure of cognitive substrates means that the relationship between the parts of one Self and that between a Self and an object of external study is a continuum, not a discrete natural kind. This suggests a key way that actual Consciousness can be studied—by becoming inherently a participant in the experiment, so as to study it from a first-person perspective. Importantly however, what happens when one fuses cognitive systems with their subject of study is that a new Self appears (a composite cognitive system), showing that the Self can remain invariant while pursuing scientific study of functional cognition and behavior (the left of the spectrum), but essentially must change in order to gain first-hand knowledge of consciousness in other cognitive systems. Images are courtesy of Jeremy Guay of Peregrine Creative.

Most surprisingly, the plasticity and capacity for bioengineering and chimerization (recombination of biological and engineered parts in novel configurations) erases the sharp divide between first person and third person perspectives. This has been a fundamental, discrete distinction ever since Descartes, but the capacity for understanding and creating new combinations shows a continuum even in this basic distinction (Figure 11). The fact that Selves are not monadic means we can share parts with our subject of inquiry. If one has to *be* a system in order to truly know what it’s like to be that system (1st person perspective), this is now possible, to various degrees, by physically merging one’s cognitive architecture with that of another system. Of course, by strongly coupling to another agent, one doesn’t remain the same and experience the other’s consciousness; instead, a new Self is created that is a composite of the two prior individuals and has composite cognition. This is why consciousness research is distinct in strong degree from other scientific topics. One can observe gauges and instruments for 3rd-person science and remain the same Self (largely; the results of the observation may introduce small alterations in the cognitive structure). However, data on 1st person experiential consciousness cannot be taken in without fundamentally changing the Self (being an effective homunculus by watching the neuroscience data corresponding to the movies inside the heads of other people is impossible for the same reason that there is no homunculus in each of our heads). The study of consciousness, whether done *via* scientific tools or *via* the mind’s own capacity to change itself, inevitably alters the Subject. Thus, standard (3rd-person) investigations of this process leave open the ancient question as to whether specific upgrades to cognition induce truly discontinuous jumps in consciousness. The TAME framework is not incompatible with novel discoveries about sharp phase transitions, but it takes the null hypothesis to be continuity, and it remains to be seen whether contrary evidence for truly sharp upgrades in consciousness can be provided. Future, radical brain-computer interfaces in human patients are perhaps one avenue where a subject undergoing such a change can convince themselves, and perhaps others, that a qualitative, not continuous, change in their consciousness had occurred.

With respect to the question of *consciousness per se*, as opposed to neural or behavioral correlates of consciousness, we have one major functional tool: general anesthesia. It is remarkable that we can readily induce a state in which all the individual cells are fine and healthy, but the larger Self is simply gone [although, some of the parts can continue to learn during this time (Ghoneim and Block, 1997)]. Interestingly, general anesthetics are gap junction blockers (Wentlandt et al., 2006): consistent with the cognitive scaling example above, shutting down electrical communication among the cells leads to a disappearance of the higher-level computational layer while the cellular network is disrupted. GJ blockers are used to anesthetize living beings ranging across plants, Hydra, and human subjects (Gremiaux et al., 2014). It is amazing that the same Self (with memories and other properties) returns, when the anesthetic is removed. Of course, the Self does not return immediately, as shown by the many hallucinatory (Saniova et al., 2009; Kelz et al., 2019) experiences of people coming out of general anesthesia—it takes some time for the brain to return to the correct global bioelectric state once the network connections are allowed again (meta-stability) (Rabinovich et al., 2008). Interestingly, and in line with the proposed isomorphism between cognition and morphogenesis, gap junction blockade has exactly this effect in regeneration: planaria briefly treated with GJ blocker regenerate heads of other species, but eventually snap out of it and remodel back to their correct target morphology (Emmons-Bell et al., 2015). It is no accident that the same reagents cause drastic changes in the high-level Selves in both behavioral and morphogenetic contexts: evolution uses the same scheme (GJ-mediated bioelectrical networks) to implement both.

The epistemic problem of Other Minds has been framed to imply that we cannot directly ever be sure how much or what kind of consciousness exists in any particular system under study. The TAME framework reminds us that this is true even for components of ourselves (like the non-verbal brain hemisphere). Perhaps the confabulation system enables one part of our mind to estimate the agency of other parts (the feelings of consciousness and free will) and develop models useful for prediction and control, applying in effect the empirical criteria for persuadability internally. The ability to develop a “theory of mind” about external agents can readily be turned inward, in a composite Self.

Are all cognitive systems conscious? The TAME framework is compatible with several views on the nature of consciousness. However, the evolutionary conservation of mechanisms between brains and their non-neural precursors has an important consequence for the question of where consciousness could be found. To the extent that one believes that mechanisms in the brain enable consciousness, all of the same machinery and many similar functional aspects are found in many other places in the body and in other constructs. TAME emphasizes that there is no principled way to restrict consciousness to “human-like, full-blown sophisticated brains,” which means one has to seriously consider degrees of consciousness in other organs, tissues, and synthetic constructs that have the same features neurons and their networks do (Trewavas and Baluska, 2011; Baluska et al., 2016, 2021; Baluska and Reber, 2019). The fundamental gradualism of this framework suggests that whatever consciousness is, some variant and degree thereof has to be present very widely across autopoietic systems. TAME is definitely incompatible with binary views that cut off consciousness at a particular sharp line and it suggests no obvious way to define cognitive systems that have no consciousness whatsoever. A big open question is whether the continuum of cognition (and consciousness) contains a true “0” or only infinitesimal levels for very modest agents. One is tempted to imagine what properties a *truly minimal* agent would have to have; not being fully constrained by local forces, and ability to pursue goals, both seem key, and both of these are present to a degree in even single particles (*via* quantum indeterminacy and least action behavior). The type and degree of scaling (or lack thereof) of these capacities in bulk inorganic matter vs. highly-organized living forms is a fertile area for future development of TAME and will be explored in forthcoming work.

## Conclusion

### A More Inclusive Framework for Cognition

Regenerating, physiological, and behaving systems use effort (energy) to achieve defined, adaptive outcomes despite novel circumstances and unpredictable perturbations. That is a key invariant for cognition; differences in substrate, scale, or origin story among living systems are not fundamental, and obscure an important way to unify key properties of life: the ability to deploy intelligence for problem-solving in diverse domains. Modern theories of Mind must eventually handle the entire option space for intelligent agents, which not only contains the familiar advanced animals we see on Earth, but can also subsume ones consisting of radically different materials, ones created by synthetic bioengineering or combinations of evolution and rational design in the lab, and ones of exobiological as well as possible terrestrial origins. The advances of engineering confirm and put into practice an idea that was already entailed by evolution: that cognitive traits, like all other traits, evolved from humbler variants, forming a continuum. There are no biologically-valid binary categories in this space. Take the prevalent legal definition of human “adults,” who snap into being at the age of 18; such binary views on cognitive properties are fictitious coarse-grainings useful for our legal system to operate, but no more than that. There is no bright line between “truly cognitive” and “pseudo cognitive” that can ever be drawn between two successive members of an evolutionary lineage. The error of “committing Anthropomorphism” is a pseudo-scientific “folk” notion useful for only the most trivial examples of failure to scale down complex claims proportionally to simpler systems; engineering requires us to determine what level of cognitive model enables the most fruitful prediction and control.

Every intelligence is a collective intelligence, and the modular, multi-scale architecture of life means that we are a holobiont in more than just the sense of having a microbiome (Chiu and Gilbert, 2015)—we are all patchworks of overlapping, nested, competing, and cooperating agents that have homeostatic (goal-directed) activity within their self-constructed virtual space at a scale that determines their cognitive sophistication. A highly tractable model system for unconventional cognition, in which these processes and the scaling of Selves can not only be seen but can also be manipulated, is morphogenetic homeostasis. The process of construction and remodeling (toward anatomical features) of cellular collectives shows crucial isomorphism to cognitive aspects of the many-into-one binding like credit assignment, learning, stress reduction, etc. The partial wiping of ownership information on permanent signals makes gap junctional coupling an excellent minimal model system for thinking about biological mechanisms that scale cognition while enabling co-existence of subunits with local goals (multiple levels of overlapping Selves, whose scale and borders are porous and can change during the lifetime of the agent). However, many other substrates can no doubt fulfill the same functions.

### Next Steps: Conceptual and Empirical Research Programs

The TAME framework is conceptually incomplete in important ways. On-going development is proceeding along lines including merging with other frameworks such as Active Inference (Friston, 2013; Badcock et al., 2019; Ramstead et al., 2019), Rosen’s (M,R) and Anticipatory Systems (Rosen, 1973, 1979, 1985; Nasuto and Hayashi, 2016), and recent advances in information theory as applied to individuality and scaling of causal power (Hoel et al., 2013, 2016; Krakauer et al., 2014; Daniels et al., 2016). It will be critical to more rigorously develop the waypoints along the Persuadability Continuum, including understanding of what an “increased capacity” human (or non-human) would be like, in contrast to the “diminished capacity” with which we are well familiar from legal proceedings [the right side of the continuum, corresponding to radically expanded cognitive light cones (Śāntideva Bstan ’dzin rgya m and Comité de traduction Padmakara, 2006)].

The TAME framework suggests numerous practical research directions immediately within reach (some of which are already pursued in our group), including developing biomedically-actionable models of morphogenetic plasticity and robustness as meta-cognitive error correction mechanisms, tissue training paradigms for anatomical and physiological outcomes, exploiting learning properties of pathway models for regenerative medicine (Herrera-Delgado et al., 2018; Biswas et al., 2021), and creation of AI platforms based on multi-scale agency architectures that do not rely on neuromorphic principles.

### Beyond Basic Science: Up-to-Date Ethics

The TAME framework also has implications for ethics in several ways. The current emphasis for ethics is on whether bioengineered constructs (e.g., neural cell organoids) are sufficiently like a human brain or not (Hyun et al., 2020), as a criterion for acceptability. Likewise, existing efforts to extend ethics focus on natural, conventional evolutionary products such as invertebrates (Mikhalevich and Powell, 2020). TAME suggests that this is insufficient, because many different architectures for cognition are possible (and will be realized)—similarity to human brains is too parochial and limiting a marker for entities deserving of protection and other moral considerations. We must develop a new ethics that recognizes the diversity of possible minds and bodies, especially since combinations of biological, engineered, and software systems are, and increasingly will be, developed. What something looks like and how it originated (Levin et al., 2020; Bongard and Levin, 2021) will no longer be a good guide when we are confronted with a myriad of creatures that cannot be comfortably placed within the familiar Earth’s phylogenetic tree.

Bioengineering of novel Selves raises our moral responsibility. For eons, humans have been creating and releasing into the world advanced, autonomous intelligences—*via* pregnancy and birth of other humans. This, in Dennett’s phrase, has been achieved until now *via* high levels of “competency without comprehension” (Dennett, 2017); however, we are now moving into a phase in which we create beings *via* comprehension—with rational control over their structure and cognitive capacities, which brings additional responsibility. A new ethical framework will have to be formed without reliance on binary folk notions such as “machine,” “robot,” “evolved,” “designed,” etc., because these categories are now seen to not be crisp natural kinds. Instead, wider approaches (such as Buddhist concern for all sentient beings) may be needed to act ethically with respect to agents that have preferences, goals, concerns, and cognitive capacity in very unfamiliar guises. TAME seeks to break through the biases around contingent properties that drive our estimates of who or what deserves proper treatment, to develop a rational, empirically-based mechanism for recognizing Selves around us.

Another aspect of ethics is the discussion of limits on technology. Much of it is often driven by a mindset of making sure we don’t run afoul of the risks of negative uses of specific technologies (e.g., genetically-modified organisms in ecosystems). This is of course critical with respect to the new bioengineering capabilities. However, such discussions often are one-sided, framed as if the *status quo* was excellent, and our main goal is simply to not make things worse. This is a fundamental error which neglects the opportunity cost of failing to fully exploit the technologies which could drive advances in the control of biology. The *status quo* is not perfect—society faces numerous problems including disparities of quality of life across the globe, incredible suffering from unsolved medical needs, climate change, etc. It must be kept in mind that along with the need to limit negative consequences of scientific research, there is a *moral imperative* to advance aspects of research programs that will (for example) enable the cracking of the morphogenetic code to revolutionize regenerative medicine far beyond what genomic editing and stem cell biology can do alone (Levin, 2011).

The focus on risk arises from a feeling that we should not “mess with nature,” as if the existing structures (from anatomical order to ecosystems) are ideal, and our fumbling attempts will disrupt their delicate balance. While being very careful with powerful advances, it must also be kept in mind that existing balance (i.e., the homeostatic goals of systems from cells to species in the food web) was not achieved by optimizing happiness or any other quality commensurate with modern values: it is the result of dynamical systems properties shaped by the frozen accidents of the meanderings of the evolutionary process and the harsh process of selection for survival capacity. We have the opportunity to use rational design to do better than the basic mechanisms of evolution allow.

Importantly, current technologies are forcing us to confront an existential risk. Swarm robotics, Internet of Things, AI, and similar engineering efforts are going to be creating numerous complex, goal-driven systems made up of competent parts. We currently have no mature science of where the goals of such novel Selves come from. TAME reminds us that it is essential to understand how goals of composite entities arise and how they can be predicted and controlled. To avoid the Skynet scenario (Bostrom, 2015), it is imperative to study the scaling of cognition in diverse substrates, so that we can ensure that the goals of powerful, distributed novel beings align with ours.

Given the ability of human subunits to merge into even larger (social) structures, how do we construct higher-order Selves that promote flourishing for all? The multicellularity-cancer dynamic (Figure 9) suggests that tight functional connections that blur cognitive boundaries among subunits is a way to increase cooperation and cognitive capacity. However, simply maximizing loss of identity into massive collectivism is a well-known failure at the social level, always resulting in the same dynamic: the goals of the whole diverge sharply from those of the parts, which become as disposable to the larger social Self as shed skin cells are to us. Thus, the goal of this research program beyond biology is the search for optimal binding policies between subunits, which optimize the tradeoffs needed to maximize individual goals and well-being (preserving freedom or empowerment) while reaping the benefits of a scaled-up Self at the level of groups and entire societies. While the specific binding mechanisms used by evolution are not guaranteed to be the policies we want at the social level, the study of these are critical for jump-starting a rigorous program of research into possible ways of scaling that could have social relevance. These issues have been previously addressed in the context of evolutionary dynamics and game theory (Maynard Smith and Szathmáry, 1995; Michod and Nedelcu, 2003; Van Baalen, 2013), but can be significantly expanded using the TAME framework.

In the end, important ethical questions around novel agents made of combinations of hardware, software, evolved, and designed components always come back to the nature of the Self. The coherence of a mind, along with its ability to pursue goal-directed activity, is central to our notions of moral responsibility in the legal sense: diminished capacity, and soon, enhanced capacity, to make *choices* is a pillar for social structures. Mechanist views of cause and effect in the neuroscience of behavior have been said to erode these classical notions. Rather than reduce Selves (to 0, in some eliminativist approaches), TAME (Levin, 2022) finds novel Selves all around us. We see more agency, not less, when evolution and cell biology are taken seriously (Levin and Dennett, 2020). The cognitive Self is not an illusion; what is an illusion is that there is only one, permanent, privileged Self that has to arise entirely bottom-up through the hill-climbing process of evolution. Our goal, at the biomedical, personal, and social levels should not be to destroy or minimize the Self but to recognize it in all its guises, understand its transitions, and enlarge its cognitive capacity toward the well-being of other Selves.

## Data Availability Statement

The original contributions presented in the study are included in the article/supplementary material, further inquiries can be directed to the corresponding author.

## Author Contributions

ML developed all the ideas and wrote the entire manuscript.

## Author Disclaimer

The opinions expressed in this publication are those of the author(s) and do not necessarily reflect the views of the John Templeton Foundation.

## Conflict of Interest

The author declares that the research was conducted in the absence of any commercial or financial relationships that could be construed as a potential conflict of interest.

## Publisher’s Note

All claims expressed in this article are solely those of the authors and do not necessarily represent those of their affiliated organizations, or those of the publisher, the editors and the reviewers. Any product that may be evaluated in this article, or claim that may be made by its manufacturer, is not guaranteed or endorsed by the publisher.

## Glossary

The following definitions of terms used in this paper (in alphabetical order) represent ways of thinking about specific terminology in the context of the proposed TAME framework. These terms have many definitions in other frameworks and are tightly interwoven, and it is likely impossible to do them full justice at this point in time (or provide uncontroversial definitions that everyone will agree capture everything of importance). Moreover, much like a theorem and its component statements, the utility of these highly-related concepts is maximized by the entire set taken together, not by crisp demarcations of any one term. The below definitions are not claimed to be uniquely correct, but merely useful; this field is still sufficiently young with respect to very basic questions, which excessively sharp definitions can limit more than they enable.

• Agency—a set of properties closely related to decision-making and adaptive action which determine the degree to which optimal ways to relate to the system (in terms of communication, prediction, and control) require progressively higher-level models specified in terms of scale of goals, stresses, capabilities, and preferences of that System as an embodied Self acting in various problem spaces. This view of agency is related to those of autopoiesis (Maturana and Varela, 1980) and anticipatory systems (Rosen, 1985).
• Consciousness—the first-person phenomenal experience of any Self—that which makes *my toothache* irreducibly different to me than anyone else’s toothache or third-person descriptions of toothaches. The degree and content of consciousness is “what it is like” to be that Self, as opposed to studying it from the outside, whether or not the Self is advanced enough to be able to verbalize it or to think about it (Nagel, 1974). Consciousness here is not meant to necessarily indicate advanced, reflexive, verbal self-consciousness but rather the basal sentience (sense-process-respond loop) which is taken to be a continuum. Moreover, because all cognitive agents are inevitably made of parts, we are all collective intelligences in a strong sense (Schwitzgebel, 2015)—what it is like to be you is exactly what it’s like to be a (particularly organized) collection of cells.
• Cognition—all of the activities undertaken by a Self, at whatever scale and of whatever material implementation, that underlie its gathering, processing, and acting on information for the purposes of adaptive action and perdurance against dissipation. Components include active inference, learning, and basal goal-directed activity, as well as complex cognitive skills such as symbolic reasoning, composition of concepts, language, and meta-cognition.
• Decision—an event during the traversal of some relevant space by a system’s state which is efficiently modeled as a choice between diverse options. The degree of “decision-making” of any given system is proportional to the spatio-temporal and complexity distance between the events that eventually gave rise to a specific outcome and the outcome itself. Advanced Selves have inputs to their decision-making machinery that are counterfactual future states. The scale at which one defines appropriate inputs (stimuli) to a system is whatever scale is most efficient for understanding the resulting decisions (Noble, 2012; Pezzulo and Levin, 2016; Flack, 2017).
• Mind—the functional, dynamic aspect of a Self that results from all of its cognitive and somatic activities, which represents the propensities for certain types of actions and possesses some degree of sentience as a first-person perspective that perdures across changes in the material components of the body.
• Intelligence—the functional ability to solve problems in various spaces (not necessarily in 3D space), not tied to specific implementations, anatomical structures, or time scales. The degree of intelligence (IQ) is proportional to competency in navigating these spaces, including especially the ability to identify paths that temporarily lead further from the goal state but eventually enable better results. Advanced intelligence exploits additional levels of self-modeling which enables multiple levels of virtual modeling of the Self and its outside world (counterfactual thought), anxiety, and creativity (identifying opportunities, as opposed to only solving problems existing right now). In particular, by focusing on the functional aspects of intelligence, and by recognizing that there is no intelligent agent that is not made of parts, Collective Intelligence is generalized here (emphasizing the architecture of functional connections between subunits) and is not viewed as a radically distinct natural kind.
• Maslow’s Hierarchy of Needs–a motivational theory of psychology that focuses on the relative types of preferences and goals which human (or other) systems pursue at various stages and scales of observation (Maslow, 1943). It also stresses degrees of integration and the modulation of higher levels by the level of stress in subunits.
• Self—a coherent system emerging within a set of integrated parts that serves as the functional owner of associations, memories, and preferences, and acts to accomplish goals in specific problem spaces where those goals belong to the collective and not to any individual sub-component. Selves are defined by the spatio-temporal scale and nature of the types of goals they can pursue—their “cognitive light cone.” They have functional boundaries and material implementations but are not identical with any specific type of substrate, and can overlap within other Selves at the same, higher, and lower-level Selves. A Self is a theoretical construct posited by external systems (such as scientists, engineers, and conspecifics) and by systems themselves (*via* internal self-models), which facilitates prediction and adaptive behavior by serving as an efficient, high-level target for intervention and control strategies.
• Stress—a system-level state which serves as a driver for homeostatic loops (operating over a variable that is progressively reduced as activity gets the system closer to its desired region of action space). The spatio-temporal and complexity scale of events that can possibly stress a system are a good indicator of that system’s cognitive sophistication. Stress can arise *via* discord between external states and the Self’s needs, between sensory stimuli and expectations, or between the goals of multiple subsystems within an agent, either within or across levels of organization. Thus, geometric frustration (Sadoc and Mosseri, 2007) and material scientists’ notions of stress as a high-level determinant of system behavior over time (Batterman and Rice, 2014; Batterman, 2015) are minimal examples of the fundamental concept of Stress, on the same continuum as metabolic stress in bacteria, competing cellular alignment forces in planar polarity of tissues, and “true psychological stress” in organisms.
